# Rapid human-driven undermining of atoll island capacity to adjust to ocean climate-related pressures

**DOI:** 10.1038/s41598-019-51468-3

**Published:** 2019-10-22

**Authors:** Virginie K. E. Duvat, Alexandre K. Magnan

**Affiliations:** 1 UMR LIENSs 7266, La Rochelle University-CNRS – 2 rue Olympe de Gouges, 17000 La Rochelle, France; 2 Institute for Sustainable Development and International Relations – Sciences Po, 27 rue Saint Guillaume, 75007 Paris, France

**Keywords:** Environmental impact, Natural hazards

## Abstract

Most studies addressing the future of atoll islands focused on ocean-climate drivers of risk, especially sea-level rise, and disregarded the role of local human disturbances. However, the future habitability of these countries will critically depend on the response of inhabited and exploited islands to ocean-climate pressures. Here, using the Maldives as a case study and based on a database including 608 islands (representing 56.8% and 86.0% of the country’s land area and population, respectively), we assess the influence of human disturbances on island natural response capacity over the last decade. We show that over the last decade, island change was rapid and primarily controlled by anthropogenic drivers. The great majority of inhabited and exploited islands now exhibit an altered-to-annihilated capacity to respond to ocean-climate pressures, which has major implications for future research and adaptation strategies. First, future studies should consider not only climate, but also anthropogenic tipping points (in contrast to climate tipping points). Second, adaptation strategies must be implemented without delay, despite climate uncertainties, in order to contain any additional detrimental path-dependency effects. This study provides critical information for better addressing the attribution issue under climate change, and a replicable rapid assessment frame.

## Introduction

**Figure 1:**
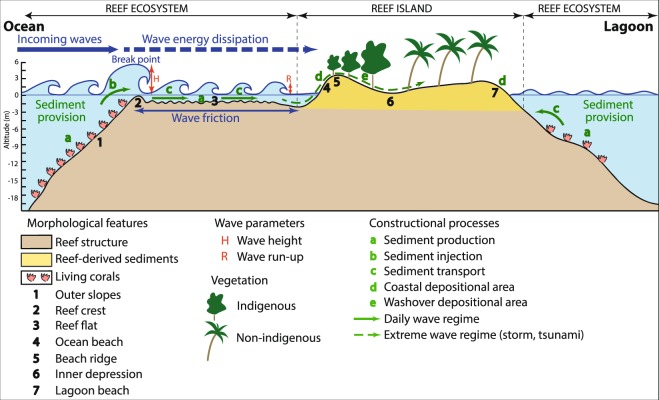
Conceptual diagram of atoll island natural dynamics showing the coastal protection services provided by the reef ecosystem. This diagram illustrates the physical processes controlling the natural dynamics of the atoll reef-island system, characterised by the high dependency of the reef island (4 to 7) on the reef ecosystem (1 to 3). The main hydrodynamic processes involved include wave friction over the reef flat, which reduces wave energy and decreases wave run-up (R), i.e. wave-driven water level at the coast. Wave run-up controls washover (e), i.e. the extent to which waves penetrate into inner land areas, especially during storms. These hydrodynamic processes drive sediment production (a), injection (b), and transport (c), from the reef to the island, including sediment deposition at the coast (d) and in inland areas (e). Any change in hydrodynamic processes would therefore result in changes in island configuration, i.e. land area, volume and elevation.

Recent studies on atoll islands’ future habitability have reaffirmed the threats posed by sea-level rise and increased wave heights to people, housing and critical infrastructure (i.e. airports, harbours and roads), and fresh groundwater supply^1–9^. Water levels and wave energy at the coast are expected to increase, which will aggravate flooding and associated damage. These impacts will be exacerbated where sea-level rise will outstrip vertical accretion rates of corals, i.e. on islands having narrow and deep reef flats with low coral cover, and/or experiencing rapid human-driven coral degradation, and/or severely affected by ocean warming and acidification^1,3,10–16^. On these islands, sea-level rise, increased wave height and reef decline will alter the coastal protection services provided by the reef ecosystem to human societies, including *(i)* the reduction of marine inundation and coastal erosion by wave energy attenuation through wave friction over the reef flat, and *(ii)* sediment delivery to the island, which is controlled by reef productivity, reef-to-shore sediment transport and the maintenance of accommodation space for sediment deposition at the coast^17–21^ (Fig. 1). The alteration of these services would result in important sediment reorganisation, and thereby in substantial changes to atoll island volume and elevation^1,3,22^. In the long run, this would cause island contraction, and eventually, disappearance. To date, a global assessment including 709 islands of the Pacific and Indian Oceans from 30 different atolls highlighted limited areal and positional changes over the past decades, notably emphasizing that 73.1% of islands were stable in area, while respectively 15.5% and 11.4% increased and decreased in size^23^. However, the critical ocean climate-related thresholds at which atoll islands may experience physical destabilisation are still incompletely understood.

The majority of the studies addressing atoll islands’ future have focused on ocean climate-related drivers of risk, based on the implicit assumption that these islands still and will still behave naturally (Fig. 1). However, previous studies have showed that inhabited and exploited islands (e.g. resort islands) generally do experience human disturbances (e.g. sediment mining, sediment transport disruption and coral reef degradation) that have already altered their natural dynamics, and as a result, exacerbated coastal erosion and marine inundation^10,24–29^. Yet, the future of atoll nations –that is, whether their populations will be able to stay or be forced to migrate abroad– will undoubtedly depend on the behaviour of their inhabited and exploited islands. More broadly, atoll territories’ future will be driven by a complex interplay of climatic, ecological and human-driven processes, to which the alteration of the reef ecosystem protection services by human activities is central. To date, however, no country-wide assessment of the extent of such human disturbances and of their implications on these services has been undertaken.

This paper addresses this gap, using the case of the most densely-populated atoll country in the world, the Maldives Islands, which host 402,071 inhabitants on a total land area of 227.4 km^2^ ^30^. It is based on an extended sample of 608 islands (out of a total of 1,149) from 23 out of the 25 atolls and oceanic reef platforms forming the country, and includes 107 (out of a total of 188) inhabited islands. For each island, we documented recent (that is, for the 2004–06 and 2014–16 periods) change in human footprint and in human pressure exerted on the reef-island environment, that is shoreline and reef flat. In line with some previous studies e.g.^31^, the human footprint, which we use as a proxy of human development, corresponds to the spatial extent of human activities, including buildings and infrastructure, facilities, roads and tracks, agriculture, industrial activities, etc., on the island scale. Unlike impact-based approaches (see e.g.^32,33)^, we do not establish any statistical correlation between human footprint and the human pressure exerted on the reef-island system.

This approach allows discussing (1) the rapidity and intensity of human-driven changes, and their detrimental effects on atoll island natural dynamics and capacity to adjust to ocean climate-related pressures; (2) the high diversity of island vulnerability profiles; and (3) the reaching of “anthropogenic tipping points”. In the context of this study, the latter are defined as human-driven critical thresholds in the reef-island system that, when exceeded, lead to a significant change in the state of the system and induce a major shift in the associated coastal protection services, often in an irreversible way. The paper concludes on important implications for future research and for the design of context-specific adaptation strategies in atoll countries and territories.

## Results

The results presented below exclude the no data islands (n.d.), the number of which varies depending on variables, but represents from 2% to 5% of the island samples considered (i.e. total, inhabited, or inhabited and exploited islands samples). The results are supported by Supplementary Material (S1 to S7), including the original database and kmz file showing study island location.

### Marked increase in human footprint

From 2006 to 2014, the total population of the sample islands grew from 226,357 to 266,812 inhabitants, representing a slightly higher rate than the national one (+17.9% against 15.1%; 30). At the national level, population growth is mostly related to the still ending phase of the demographic transition^30^, rather than to the migration of foreigners (around 15.0% of the total population in both 2006 and 2014). In addition, over the same period, the annual number of international tourists doubled (from 601,923 to 1,204,857) and the island resort bed capacity increased by a third (from 17,712 to 24,031).

Seventy-one percent of the 107 inhabited islands considered in this study experienced population growth, at a rate exceeding 5% for 59.8% of them, and even >25% for 18.6% of them. As a result, the human footprint increased through the spatial expansion of human occupancy on 47 already inhabited or exploited islands and through the new settlement or exploitation of 56 islands (S1 Table B). The development of the latter led to a decrease in the proportion of natural islands, from 374 (61.5% of the island sample) in 2004–06 to 327 (53.8%) in 2014–16. This means that 288 out of the 608 islands considered here were either inhabited or exploited (tourism, agriculture, small industry…) in 2014–16. Only 11 islands (1.8%) exhibited a contraction of human occupancy, including 9 islands that were completely abandoned following the 2004 Indian Ocean tsunami –e.g. Kandholhudoo (No16 in the database, Raa Atoll) population was first hosted on Ungoofaaru island (No71, Raa Atoll) and then relocated on the newly inhabited Dhuvaafaru island (No67, Raa Atoll)– or in the frame of the 2009 Population Consolidation Programme^34^ partly aiming at decentralising the population –e.g. Faridhoo, Maavaidhoo and Kuburudhoo populations (islands No2, No132 and No137, respectively; Haa Alifu-Noonu Atoll) were relocated on Nolhivaranfaru island (No136, Haa Alifu-Noonu Atoll).

As a result, 32.7% of the sample islands (199) exhibited a very high human footprint (i.e. >2/3 of island land area; see Materials and Methods for categorisation) in 2014–16, against 23.5% of islands (143) in 2004–06. While most of the islands that changed category moved to the next category (12 islands from ≤1/3 to 1/3 < x ≤ 2/3, and 25 islands from 1/3 < x ≤ 2/3 to >2/3), 9 islands (1.5%) experienced a dramatic increase, changing from a low (≤1/3) to a very high (>2/3) human footprint in just one decade.

**Figure 2:**
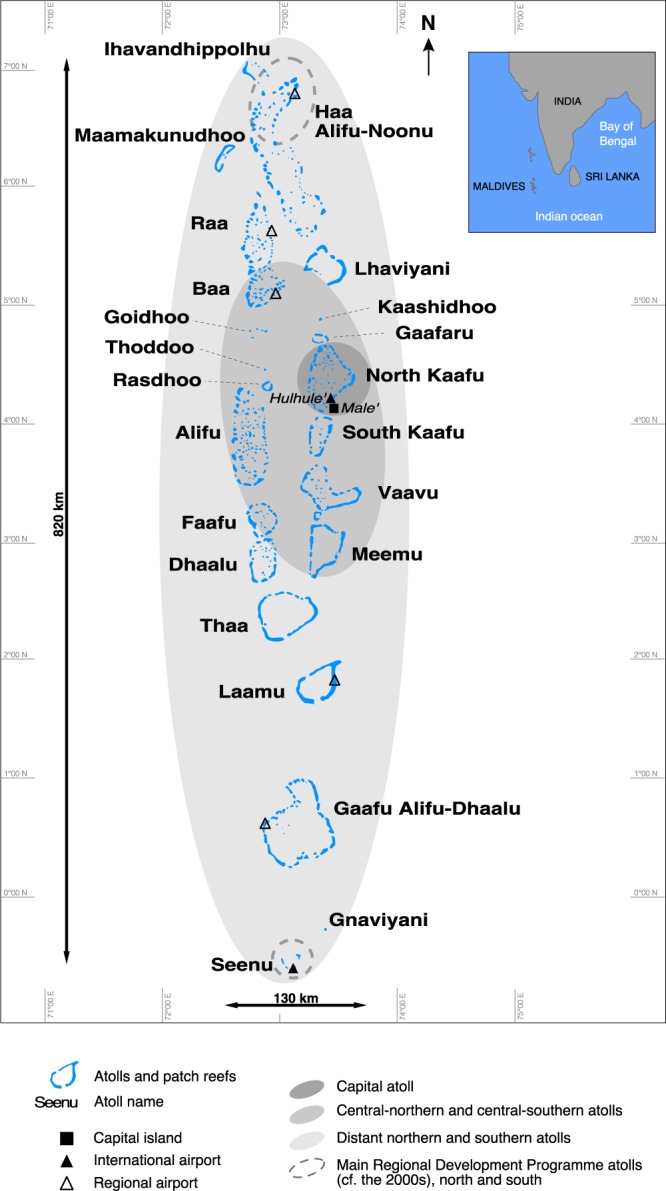
General presentation of the Maldives Islands. The capital atoll has been the historical political, population, and development centre for around 3,000 years. The central-northern and central-southern atolls have been the first ones, after the capital atoll, to benefit from the tourism-induced development boom of the 1970s/1980s. The distant northern and southern atolls represent the secondary less-developed periphery of the country that currently shows rapid socio-economic development. Two exceptions are the Haa Alifu-Noonu (northernmost) and Seenu (southernmost) atolls that have been designed in the 1990s as Regional Development Poles, and have therefore benefited from more investments than other distant atolls.

**Figure 3:**
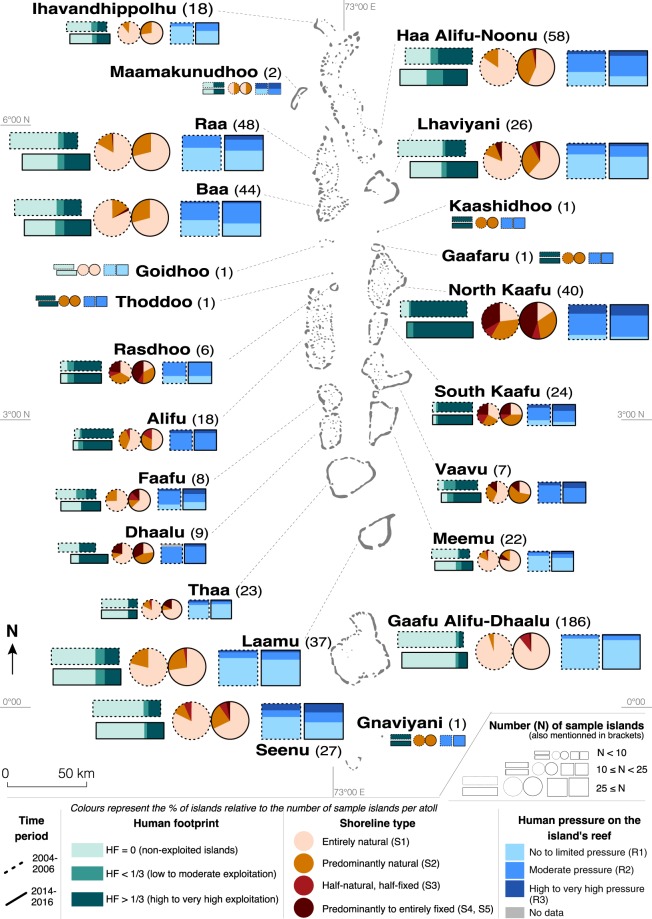
Spatial distribution of major human disturbances caused to the reef-island system in the Maldives. This map provides an overview of the results obtained. It highlights increased island occupancy (human footprint, HF), and associated increased human pressure on island shoreline (shoreline type, S) and island reef (R) in just one decade. With the exception of atolls having only one (Kaashidhoo, Goidhoo, Gaafaru, Thoddoo and Gnaviyani) or two (Maamakunudhoo) islands, the results highlight a marked increase in human pressure, even in the central atolls that already exhibited marked human disturbances in 2004–06, as a result of rapid population growth and socio-economic development.

The results highlight marked regional variability. The highest rates of change were observed in the central capital atoll of North Kaafu (Figs 2 and 3) that concentrates most of the population (especially on the capital island of Male’), infrastructures (airport island of Hulhule’) and economic activities. On this atoll, 15% of islands underwent an increase in human footprint. The central-northern (Raa, Lhaviyani, Baa) and central-southern (Meemu and Dhaalu) atolls also exhibited a marked increase in human footprint between 2004–06 and 2014–16, as illustrated by Raa, where this was the case for 31.3% of islands (Figs 3 and S1 Table A).

### Human-driven changes caused to the reef-island system

The marked increase in human footprint contributed to major changes to island and reef morphology, mainly through artificial island expansion and shoreline armouring.

#### Artificial island expansion

In just a decade, the proportion of islands having artificial reclaimed areas protruding onto their reef flat (hereafter ‘reclaimed islands’) increased by 51.2% (from 123 to 186 islands). As a result, 30.6% of the sample islands, and 64.6% of the inhabited and exploited islands, had reclaimed areas in 2014–16 (S2 Table A). From a regional perspective, the highest rates of change in the number of reclaimed islands (>50%) occurred in the distant northern (to the north of Baa) and southern (to the south of Vaavu) developing atolls, with the exception of Laamu, Lhaviyani and Seenu. In contrast, the central atolls around the capital atoll (with the exception of atolls having one single island), which already had 18.2 to 70.0% of their islands exhibiting reclaimed areas in 2004–06, showed more limited change (≤25%).

Land reclamation was generally carried out either to meet housing needs, or as a result of harbour dredging in reef flat, which encourages the reclamation of nearby reef areas through the provision of large quantities of materials. In just one decade, the number of islands having at least one proper harbour increased by 55.2% (from 96 to 149 islands), which resulted in 24.5% of the sample islands and 52.7% of the inhabited and exploited islands having a proper harbour in 2014–16 (S2 Table A).

Out of the 63 inhabited islands (i.e. 57.9%) that experienced land reclamation between 2004–06 and 2014–16, respectively 30 and 33 showed an increase in land area at rates comprised between 3 and 10% and ≥10% (S3 Table A). Among the latter, 17 islands (15.9% of the sample inhabited islands) experienced rates falling between 10 and 25%, while 16 islands (15%) showed rates >25%. Of note, changes in island land area and population size do not necessarily correlate, due to a time-lag between island expansion and the settlement of artificial areas by in-migrants.

#### Shoreline armouring

Land reclamation and harbour construction contributed to the extension of artificial shoreline (S2 Table B). While land reclamation caused the extension of longitudinal coastal structures (mostly seawalls and riprap), which were erected to stabilise newly reclaimed areas, harbour construction generally encouraged groyne construction to reduce the disturbing impact of jetties on downdrift shoreline sections affected by sediment depletion. In 2014–2016, respectively 59 (+66.3%) and 26 (+48.1%) additional islands (compared to 2004–06) had longitudinal and transversal coastal structures (S2 Table B). As a result, the proportion of islands having respectively longitudinal and transversal structures increased from 14.8% to 24.5% (representing 52.3% of the inhabited and exploited islands in 2014–16), and from 9.0 to 13.3% (representing 28.9% of the inhabited and exploited islands in 2014–16) (S2 Table B). In addition, we found that respectively 112 and 56 islands that already had longitudinal and transversal coastal structures in 2004–06 exhibited additional structures in 2014–16 (S2 Table B). Simultaneously, the number of islands having marine protection structures (mostly breakwaters) established on their reef flat increased from 21 to 45, resulting in 16.2% of the inhabited and exploited islands and 7.5% of the sample islands having such structures in 2014–16 (S2 Table B). Moreover, 35 islands that already had marine protection structures in 2004–06 had additional ones in 2014–16. The central atolls generally exhibited a relative stability in the number of islands having protection structures (most of their islands already having such structures in 2004–06) but a marked increase in the number and extent of these structures. In contrast, northern (excluding Lhaviyani) and central-southern (i.e. Vaavu, Meemu and Thaa) atolls, on which these structures were less common in 2004–06, exhibited very high rates, generally >100% (S2 Table B).

As a result of the extension of coastal protection structures along island shoreline, 66 islands (10.9% of the total sample) that had an “entirely natural” (i.e. undisturbed) shoreline in 2004–06 had at least a partially artificial shoreline in 2014–16 (S2 Table D). While 58 (9.5%) of these islands moved from the “entirely natural” to the “predominantly natural” shoreline category (See Materials and Methods), 8 islands moved to even higher categories (i.e. “half-natural, half-fixed; “predominantly fixed”; “entirely fixed”), that is, exhibited dramatic shoreline armouring. Only 4 islands (0.7%) showed a decrease in shoreline hardening. As a result, the number of islands having an “entirely natural” shoreline decreased from 472 (77.6%) in 2004–06 to 407 (66.9%) in 2014–16. In addition, the islands that already had armoured shoreline sections in 2004–06 generally experienced an extension of artificial shoreline. This was observed on 89 islands (14.6%), with 31 islands moving from category 1 (“entirely natural”) or 2 (“predominantly natural”) to categories 3 to 5 (“half-natural”, “predominantly fixed”, “entirely fixed”). As a result, some central atolls (e.g. North Kaafu and Rasdhoo) exhibited a predominant proportion of islands having a “predominantly fixed” shoreline in 2014–16. In addition, the northern and southern distant atolls exhibited a decrease in the proportion of islands having “entirely natural” shoreline and an approximately proportional increase in the number of islands having a “predominantly natural shoreline”, with these two categories taken together still representing between 44.5 and 72.2% of islands, depending on the atoll, in 2014–16 (S2 Table C and Fig. 2).

#### Pressure exerted on the island’s reef

On most atolls, the human pressure exerted on the island’s reef increased markedly over the past decade (Fig. 3). This increased pressure was mainly due to harbour basin dredging in, and sediment mining from, the island’s reef (S4 Table A). The number of islands having harbour basins dredged in reef flat increased by 61.8%, from 110 islands (18.2% of the islands sample) in 2004–06 to 178 (29.5%) in 2014–16 (S4 Table A). Additionally, some islands that already had one or more harbour basin(s) dredged in reef flat in 2004–06 experienced either harbour basin(s) extension, as observed on 38 islands (6.3%), or the establishment of one or more additional harbour basin(s), noted on 20 islands (S4 Table A). Aggregating all islands (i.e. with/without a harbour basin in 2004–06), we found that 14.5% of islands (88) experienced an increase in the number of harbour basins over the past decade (S4 Table B). This resulted in a marked extension of boat channels across island reef flat, with 77 islands (12.8%) exhibiting additional boat channels in 2014–16 compared to 2004–06 (S4 Table A).

The increase in the number of islands having harbour basins was the highest (rate ≥ 100%) in the northern (Ihavandhippolhu, Haa Alifu-Noonu, Raa, Baa), central (Rasdhoo) and distant-southern (Faafu, Dhaalu, Gaafu Alifu-Dhaalu) atolls. This highlights the effort made by the national authorities to improve transport facilities in the outer atolls in order to address the historical challenge of making outer and scattered inhabited islands accessible. In contrast, the core atolls exhibited much lower rates (14.3 to 66.7%), due to a high proportion of islands already having harbour basins in 2004–06. It is noteworthy that the establishment of additional harbour basins and the dredging of additional boat channels across island reef flat occurred on most atolls, including central (e.g. South Kaafu for harbour basins), distant (e.g. Haa Alifu-Noonu) and central-northern and -southern (e.g. Vaavu for harbour basins, Raa for boat channels) atolls (S4 Table A).

Simultaneously, 41 additional islands (+6.74% of the island sample) were affected by sediment dredging from reef flat in 2014–16 compared to 2004–06. As a result, the islands affected by this disturbance increased from 84 (13.9.% of the island sample) to 125 (20.7%). The highest rates (>100%) were observed on the two distant atolls of Haa Alifu-Noonu (from 5.2 to 19% of islands) and Vaavu (from 14.3 to 57.1% of islands) (S4 Table A).

Aggregating the data to assess change in the human pressure exerted on the island’s reef, we found that 64 islands (including most inhabited islands and representing 10.5% of the 608 sample islands) exhibited an increase, 523 islands (86.0%) a relative stability, and only 7 islands (1.2%) a decrease (S5 Table B). Twenty-eight islands (4.6% of the sample) that experienced no-to-limited (R1 in S5 Table B) or moderate (R2) human pressure on their reef in 2004–06, exhibited a dramatic increase and therefore a high to very high pressure (R3) just one decade later. As a result, 64 islands representing 10.5% of the whole sample showed a high to very high (R3) human pressure in 2014–16, while 197 islands (32.4%) and 333 islands (54.8%) showed a moderate (R2) and no-to-limited (R1) pressure, respectively (S5 Table B).

### Marked increase in disturbed island types

**Figure 4:**
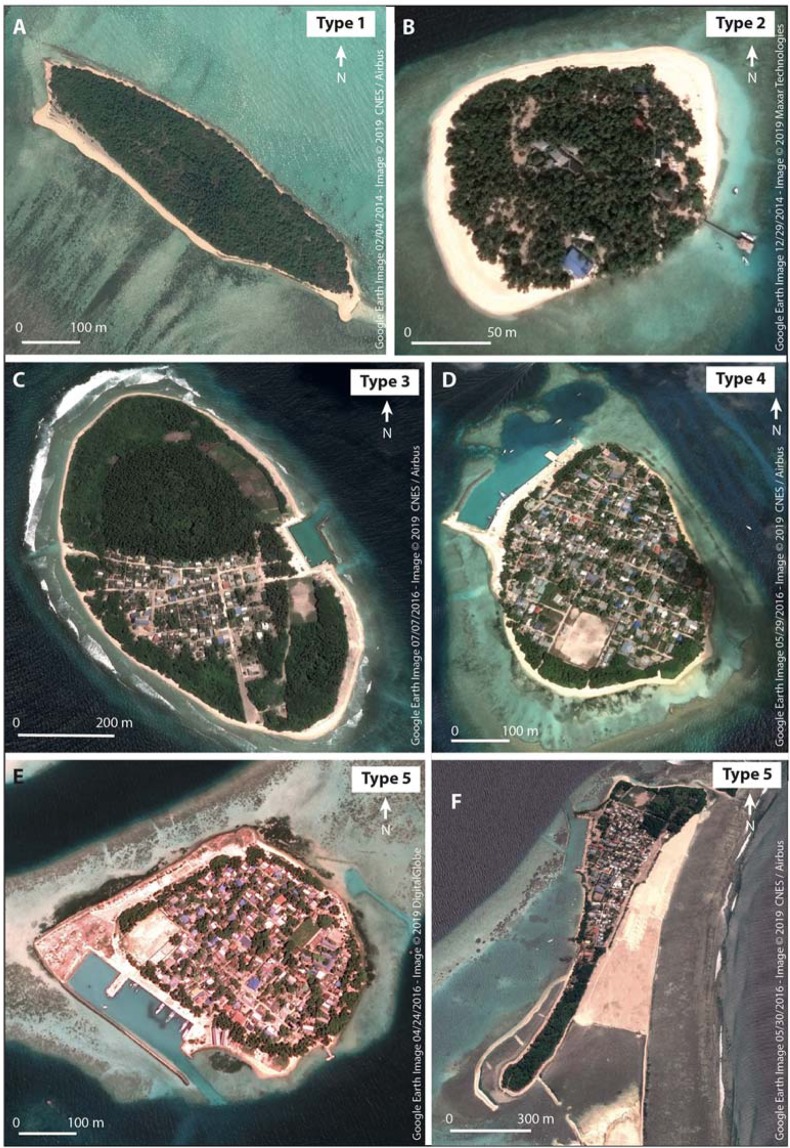
Illustration of the 5 island types highlighting the degree of human disturbance affecting the reef-island system. Panel A Maafinolhu Island (No7 in the database), Ihavandhippolhu Atoll, illustrates the situation of an “undisturbed” (here, “natural”, i.e. unexploited) island exhibiting no human disturbance (Type 1, T1). Of note, some T1 islands are exploited (e.g. nearby Manafaru Island, No8, which is a resort island). Panel B Kudabados Island (No40), North Kaafu Atoll, shows the situation of a “little disturbed island” (T2), where the construction of nearshore breakwaters along the eastern coast has modified hydrodynamics. Panel C Hirimaradhoo (No8), Haa Alifu-Noonu Atoll, is a “moderately disturbed island” (T3), where human disturbances mainly consist of the establishment of a harbour in the reef flat, which disrupts longitudinal and transversal sediment transport on the eastern side of the island, and of land reclamation on both sides of the harbour. Panel D Rasdhoo Island (No2), Rasdhoo Atoll, is a “highly disturbed island” (T4) exhibiting extensive shoreline modification and armouring, marked reef degradation and sediment transport obstruction, due to the establishment of a harbour on its northern side and to the erection of breakwaters on its eastern side. Panel E Fieeali Island (No17), Faafu Atoll, and Panel F Muli Island (No29), Meemu Atoll, are “very highly disturbed islands” exhibiting marked changes in geomorphic configuration (due to island artificial expansion, still in operation on Panel F, and leading to extensive shoreline armouring) and highly degraded reef flats (due to extensive mechanical destruction resulting from harbour establishment in, and boat channel and sediment dredging from, reef flat).

Based on the shoreline and reef disturbance indicators presented above, we classified the sample islands into five distinct categories (Fig. 4, Materials and Methods, and S5 Table C). While Types 1 and 5 (T1, T5) respectively correspond to an island exhibiting no human disturbance and to a very highly disturbed island, T2 (low disturbance), T3 (moderate disturbance) and T4 (high disturbance) illustrate intermediate gradual situations. We found that 17.6% of islands (107 out of 608) moved to a more disturbed type, while 79.3% (482 out of 608) remained in the same category (mainly T1, T2 and T3) and 0.8% (5 out of 608) moved to a less disturbed type (S5 Table D). The main changes observed were from T1 to T2 and T3 (respectively 21 and 15 islands, 3.5% and 2.5% of the island sample); from T2 to T3 (43 islands, 7.1%); and from T3 to T5 (12 islands, 2%). As a result of changes in island category, T1, T2, T3, T4 and T5 represented respectively 54.1%, 11.5%, 22.2%, 2.3% and 7.6% of the sample islands in 2014–16 (2.3% n.d.).

Furthermore, in just one decade, 46.7% of the inhabited islands (50 out of 107) moved to a more disturbed type, while 49.5% remained in the same category and 0.9% moved to a less disturbed type (+2.8% of no data islands). For these islands, the main changes observed were from T2 to T3 (24 islands, 22.4%) and from T3 to T5 (11 islands, 10.3%). As a result of changes in island category, T1, T2, T3, T4 and T5 represented respectively 0.0%, 0.9%, 68.2%, 7.5% and 20.6% (2.8% n.d.) of inhabited islands in 2014–16, meaning that 96.3% of these islands were moderately (T3) to very highly (T5) disturbed by human activities (S5 Table E). It is noteworthy that in 2004–06, these proportions were respectively of 5.6%, 24.3%, 53.3%, 7.5% and 6.5% (2.8% n.d.), making 67.3% of the inhabited islands moderately (T3) to very highly (T5) disturbed by human activities. Taken together, the T4 and T5 inhabited islands increased from 14.0% in 2004–06 to 28.0% in 2014–16.

**Figure 5:**
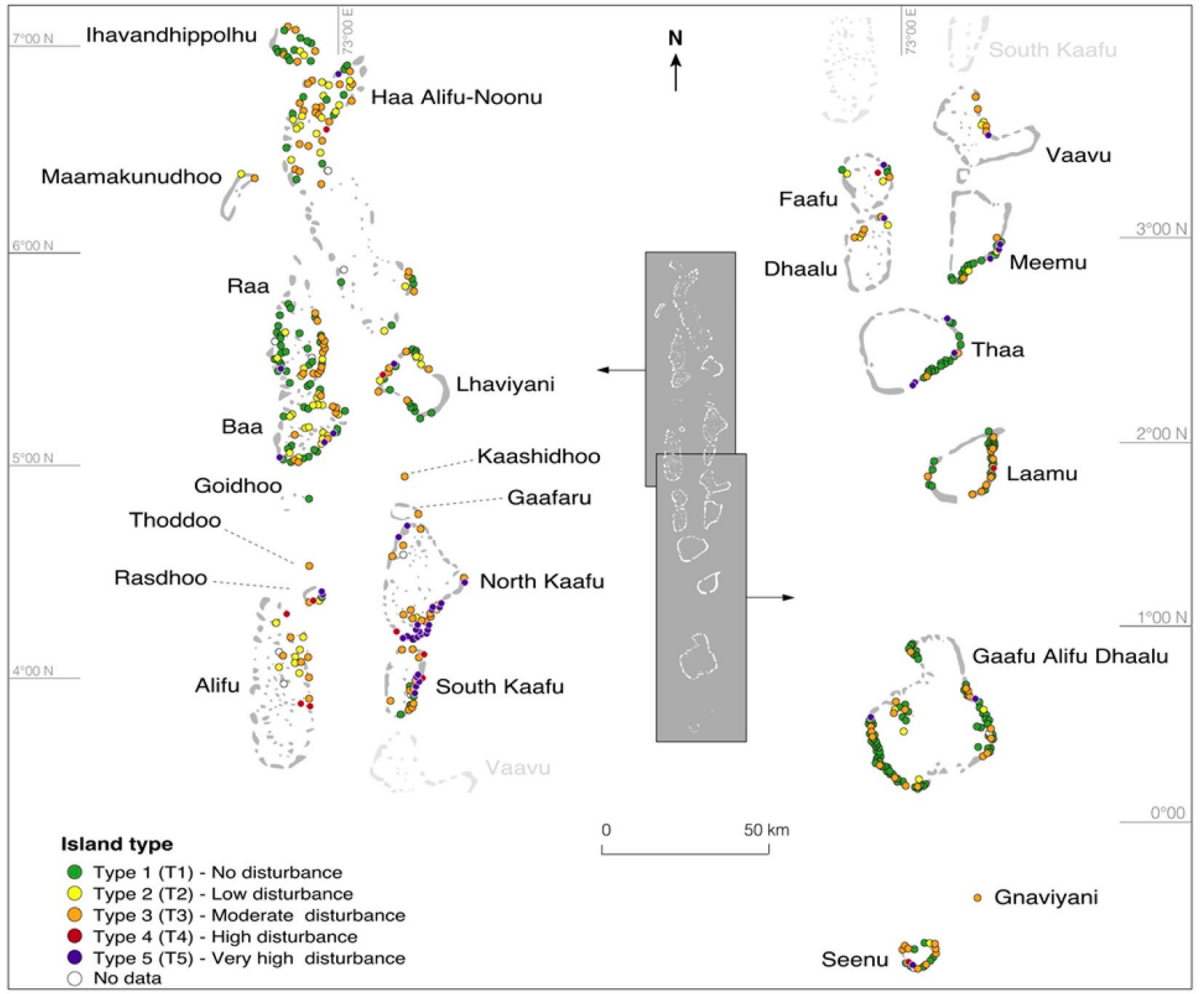
Spatial distribution of island types. T3 to T5 islands prevail in the central (i.e. densely-populated and highly-developed) atolls of North Kaafu and South Kaafu, while T1, T2 and T3 are the most represented types in distant (i.e. less populated and developed) atolls. This critically highlights the environmentally-detrimental development patterns of the Maldives Islands.

In 2014–16, T4 and especially T5 prevailed in the central atolls of North Kaafu and South Kaafu, reflecting the high human degradation of the reef-island system in these long-standing developed atolls, while T1 to T3 prevailed in the rest of the country (Fig. 5). However, the spatial distribution of island types shows that highly and very highly disturbed islands (i.e. T4 and T5) now occur on most atolls.

## Discussion

### The major role of anthropogenic control

Based on an extended dataset including 608 islands (52.9% of the Maldives Islands) from 23 atolls and oceanic reef platforms, this study constitutes the first detailed, nation-wide assessment of human-driven undermining of the coastal protection services provided by the reef ecosystem to island communities. It confirms that over the last decade, human activities have been a major driver of reef-island morphological change^35^. Increasing human pressure led, in just one decade, to the exploitation of 56 natural islands (9.2% of the sample islands) and to the expansion of human occupancy on 47 already inhabited and exploited islands (7.7%). As a result, the proportion of islands exhibiting a very high human footprint (>2/3 of island land area) increased by 9.2% (from 143 to 199 islands). Additionally, most of the islands that were already inhabited or exploited in 2004–06 underwent substantial changes, especially through artificial expansion. Around 64.6% of these islands had reclaimed areas in 2014–16, and the proportion of islands that experienced human-driven areal expansion between 2004–06 and 2014–16 increased by 51.2% (from 123 to 186 islands). More than half of these islands (33 islands) experienced high (>10%) island growth rates, with respectively 17 and 16 islands exhibiting rates falling between 10 and 25%, and >25%. Simultaneously, the proportion of islands having harbour basins dredged in reef flat increased by 61.8% (from 110 to 178 islands).

**Figure 6:**
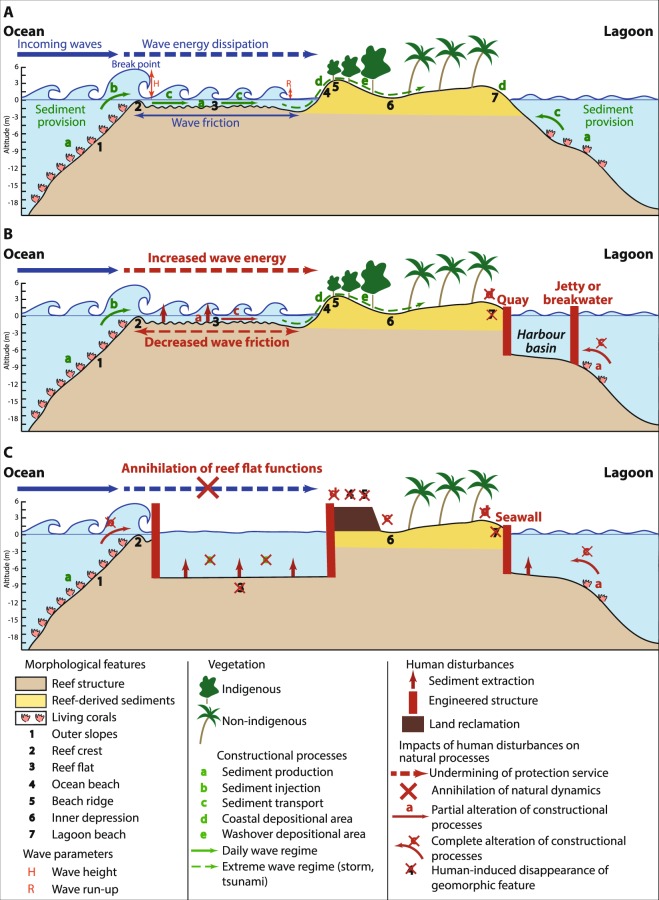
Conceptual diagram showing various degrees of human-driven undermining of atoll island natural dynamics. Panel A reflects the situation of Type 1 (T1) islands that are undisturbed by human activities. Panel B illustrates the situation of T3 islands, the lagoon side of which is generally the most disturbed, mainly by the establishment of a harbour that causes the mechanical destruction of the reef flat and obstructs longitudinal and transversal sediment transport, and by sediment dredging from their ocean-side reef flat. These disturbances cause the complete disappearance of some geomorphic features (here, the lagoon beach), reduce sediment injection into the island system (b), and alter constructional processes on the whole (a, c, d). Panel C shows the situation of T4 and T5 islands that exhibit marked changes in geomorphic configuration on their lagoon and ocean sides (disappearance of beaches, i.e. 4, 5 and 7 in the figure), and the almost-to-complete annihilation of the services (a, b and c) provided by the reef ecosystem.

Land reclamation on and harbour dredging in island reef flat have important detrimental impacts on the coastal protection services delivered by the reef-island system (Fig. 6). The direct effects of these activities include the mechanical destruction of the reef flat, which affects sediment production, and the disruption of longshore and cross-shore sediment transport. Their indirect effects include shoreline armouring (carried out to stabilise newly reclaimed areas), increased sediment mining from island reef flat (carried out to provide the materials required for land reclamation and the construction of coastal protection structures), and increased boat channel dredging across island reef flat, which affect sediment production, transfer and deposition at the coast. These results highlight the local-scale adverse consequences of rapid population growth (+15.1% between 2006 and 2014) and socio-economic development (mean GDP annual growth rate of 7.7% between 2006 and 2014) on the reef -island system.

### Scientific way forward

These results imply that assessing the control exerted by human activities on the reef-island system’s change is absolutely critical to properly understand observed trends and appreciate their implications on islands’ future. In fact, not considering that island expansion is artificial, one would erroneously conclude that island growth is indicative of a healthy (i.e. still potentially able to naturally adapt to ocean climate-related pressures) reef-island system. Quite the opposite, it is here indicative of a high degradation of the reef-island system.

Better capturing the controlling factors of change on the island scale provides a unique opportunity to discuss the attribution of observed changes (e.g. relative weights of natural vs. anthropogenic drivers), and hence provide new information for prospective modelling studies. Here, we use two major human disturbance indicators (i.e. on shoreline and reef) to develop a typology, classify more than 600 islands and highlight the most critical situations of T4 and T5 islands. Advancing knowledge further would require the scientific community to design and implement physical models integrating the respective effects of different types and degrees of human disturbance on the functionality of the reef-island system. Importantly, such a modelling effort will only be beneficial if ran for contrasting situations to account for context specificities, e.g. using our five island types.

### Tracking anthropogenic tipping points

Tipping points refer to ‘critical thresholds in a system that, when exceeded, can lead to a significant change in the state of the system, often with an understanding that the change is irreversible’^36^:262. Capturing tipping points is critical to understand abrupt changes and irreversibility in a given system’s functioning, and has considerable implications in terms of how to respond and anticipate non-linear changes. Tipping points are often discussed in the scientific literature for the physical climate system (e.g., collapse of the West Antarctica Ice Sheet and implications for global sea-level rise) or ecological systems (e.g. mid-century coral decline due to global warming), but less is known about tipping points in the geomorphic components of a given system (here, atoll island shoreline and reef) due to direct human disturbances. This describes the “anthropogenic tipping points” as discussed in this study. We argue here that the above-described island typology can help identify “anthropogenic tipping points”.

The results presented here show that the two services provided by the reef ecosystem, namely wave energy attenuation and sediment provision, which are absolutely essential to risk reduction and to the persistence of these islands and of the whole country over time, have so far been preserved from human deterioration on all T1 islands (“undisturbed islands”), which represent 54.1% of the sample islands, and only on 12.5% of the 288 inhabited and exploited islands (Fig. 6A). This means that on 43.6% of the sample islands and on 83.0% of the inhabited and exploited islands (T2 to T5, from “low disturbance” to “very high disturbance”), these critical services have to some extent been undermined by human development. In fact, 11.5% of the sample islands and 16.7% of the inhabited and exploited islands exhibit a localised and limited undermining of these services (T2), and are therefore still potentially able to naturally respond and adjust to ocean climate-related pressures through sediment reorganisation. In addition, 22.2% of the sample islands and 46.2% of the inhabited and exploited islands exhibit a partial but significant undermining of these services (T3), and are therefore not fully able to naturally respond and adjust to ocean climate-related pressures. This situation is for example illustrated by those islands that have a harbour basin annihilating reef-to-island sediment transport on their lagoon side and exhibit decreased wave energy attenuation due to sediment mining from their reef flat on their ocean side (Fig. 6B). Moreover, 9.9% of the sample islands and 20.1% of the inhabited and exploited islands exhibit a “high to very high” undermining (T4 and T5) of these services by human activities, and have therefore either almost entirely (T4) or entirely (T5) lost their natural capacity to respond and adjust to ocean climate-related pressures. These islands, which generally exhibit a predominantly-to-entirely artificial shoreline and experience an almost annihilation of longshore and cross-shore sediment transport on both their lagoon and ocean sides (Fig. 6C), are now committed to a path-dependency effect in terms of human intervention. On these islands, due to the irreversible destruction of the coastal protection services that drive island adjustment to ocean climate-related changes, an anthropogenic tipping point has been reached.

The fact that the 20.1% of inhabited and exploited islands (T4, T5) have already reached this tipping point and that 46.2% (T3) of these islands may, in regard to their trajectory of change, reach it over the coming decade(s), raises serious concerns in the context of sea-level rise (+1.3 mm/year ±0.4 and +1.4 mm/year ± 0.4 for Male’, North Kaafu Atoll, and Gan, Seenu Atoll, respectively, between 1950 and 2009^37^) and increased flooding events^38^.

### Implications for adaptation to climate change

Such results have two major implications for societal adaptation to ocean climate-related changes, especially sea-level rise and increased island flooding.

First, they reinforce the idea that the degree to which human activities have undermined atoll island capacity to respond and adjust to ocean climate-related pressures should be taken into account when designing response options and adaptation pathways. In the specific context of atoll countries and territories, this suggests that the adequate spatial scale to design well-adapted strategies is the island rather than the archipelago scale. The critical point to consider when designing an adaptation strategy for any given island should therefore first be whether this island has already or almost reached or not the anthropogenic tipping point beyond which human intervention is unavoidable to face ocean climate-related risks. With this respect, we argue that the island typology (i.e. from T1 to T5) and rapid assessment frame proposed in this paper offer a way to find a compromise between two extremities, i.e. too broad assessments conducted on the country scale and too time-consuming in-depth assessments conducted on the island scale.

Second, the highly-contrasting island types highlighted in this study imply the design of diversified adaptation strategies. For those islands that still have the natural capacity to adjust (either entire for T1, or partial for T2-T3), and that can therefore be expected to keep it over the next decades in the face of limited external ocean climate-related forcing if they were sustainably managed, preserving or restoring the coastal protection services provided by the reef ecosystem should be a key priority. Here, nature-based solutions offer a huge area of opportunity. On the contrary, it should be acknowledged that hard-engineered measures (e.g. seawalls, breakwaters) are required on those inhabited and exploited islands that have reached the above-described anthropogenic tipping point. On those islands that exhibit highly disturbed sediment production and transport patterns, together with high densities of human assets (people, buildings, etc.), the use of adequately designed and calibrated protection structures should be prioritised without further delay. With the exception of a limited number of islands on which proper engineered structures were constructed as a result of international aid or following extensive reclamation works operated by the public authorities, and like most developing island countries, the Maldives Islands mostly have handmade, small and poorly designed and built protection structures that do not constitute a climate change-proof solution^39,40^. Collectively, these points suggest that robust adaptation decisions can be taken from now on, on the island scale or at least for different island types, to limit further maladaptive path-dependency effects on the island scale, regardless climate-related uncertainties.

## Conclusion

The 608 sample islands used in this study are representative of the variability of human disturbances across atoll islands^41,42^, making the findings of relevance beyond the case of the Maldives Islands. The results provide the first nation-wide and island-scale assessment of the situation of an atoll country, especially of the inhabited and exploited islands on which the future habitability of such a country critically relies. This study notably highlights not only the major contribution of human disturbances to recent island change, but also and most importantly, that respectively 20.1% and 46.2% of the inhabited and exploited islands of the Maldives have already reached and may reach in the near future an anthropogenic tipping point beyond which island armouring will be the only solution to maintain islands in the face of sea-level rise and extremes, especially. Yet, there is high uncertainty on the future habitability of such armoured islands, which would lie below sea level and therefore likely lose not only their freshwater underground supply but also all cultivable land, due to saltwater intrusion. These results lead us to argue, first, that there is an urgent need to adequately consider the to-date disregarded anthropogenic drivers in atoll island change studies in order to not only take into account their current impacts, but also the lock-in effects induced by the human-driven undermining of the coastal protection services provided by the reef ecosystem. Second, and given the diversity of atoll island profiles, there is a need to design island- or at least island type-specific adaptation strategies. Third, given the rapidity of change observed in the Maldives Islands, we argue that such adaptation strategies must be designed and implemented without delay, despite climate uncertainties, in order to contain any additional detrimental path-dependency effects.

## Materials and Methods

### Presentation of the island sample

The total number of islands in the Maldives is constantly changing, due to the highly unstable character (i.e. disappearance and reformation) of the smallest islands. Here, we used the statistics generated by Naseer and Hatcher^43^, which constitute the most precise dataset documenting the physical characteristics of the archipelago. We therefore consider the total number of Maldivian islands to be 1,149. The 608 sample islands considered in this study therefore represent 52.9% of the Maldives Islands and are distributed among 23 atolls and oceanic reef platforms out of the 25 inventoried (Alifushi and Vattaru, which have one single island, are not documented in this study). These 608 islands are the islands for which the 2004–06 and 2014–16 satellite images provided by Google Earth and used in this study were available and treatable. The island sample considered for each atoll and oceanic reef platform represents 16.7% to 100.0% of islands, depending on the case (Table 1). Of note, the number of documented islands changes from the first to the second date for some variables, due first to variations in image treatability, and second to the aggregation (either natural, or human-induced) of 11 islands between 2004–06 and 2014–16. No data (n.d.) does not exceed 5% of the sample, whatever the variable considered.

**Table 1:** Significance and main characteristics of the island sample considered in this study per atoll.

Atoll or patch reef name	Total island land area (km^2^)**	Total estimated number of islands**	Significance of sample islands		Population size in 2014***
			Number	% of the total number of islands	
Ihavandhippolhu	5.7	24	18	75	57,078
Haa Alifu-Noonu	68.7	164	58	35.4	
Maamakunudhoo	1.0	4	2	50.0	
Raa	12.9	92	48	52.2	15,819
Lhaviyani	7.2	55	26	47.3	8,380
Baa	5.5	64	44	68.7	9,601 (includes Goidhoo)
Kaashidhoo*	2.9	1	1	100.0	Included in North and South Kaafu
Goidhoo	2.2	6	1	16.7	Included in Baa
Gaafaru	0.2	1	1	100.0	Included in North and South Kaafu
North Kaafu	9.4	56	40	71.4	167,996 (total North and South Kaafu)
Thoddoo*	1.6	1	1	100.0	Included in Alifu
Rasdhoo	0.6	10	6	60.0	Included into Baa
Alifu	8.3	81	18	22.2	15,561
South Kaafu	2.0	30	24	80.0	Included in North Kaafu
Vaavu	0.9	29	7	24.1	1,811
Faafu	2.2	22	8	36.4	4,365
Meemu	4.2	39	22	56.4	5,022
Dhaalu	4.4	45	9	20.0	5,786
Thaa	9.3	70	23	32.8	9,656
Laamu	23.1	78	37	47.4	12,676
Gaa Alifu-Dhaalu	34.3	246	186	75.6	21,911
Gnaviyani*	5.1	1	1	100.0	8,510
Seenu	15.0	27	27	100.0	21,275

*Are oceanic reef platforms. **Based on Naseer and Hatcher, 2004^43^. ***Excludes the population of resort islands (28,367 people), and of industrial and other uninhabited islands (8,257 people).

### Definitions of the terms used in this study

- ‘Human footprint’ corresponds to the degree of human occupancy for a given island. It corresponds to the proportion of the island exhibiting buildings and infrastructure, facilities, roads and tracks, agriculture, industrial activities, inland vegetation removal. It excludes unexploited and ‘natural’, i.e. non-cultivated vegetated areas.
- A ‘natural island’ (e.g. Ihavandhippolhu Atoll, Nos4, 5,19) is defined as an island having no permanent human construction currently in use and exhibiting no large-scale sign of human exploitation of terrestrial resources. Such an island may have been inhabited in the past and then depopulated, as observed for a number of islands since the 2004 tsunami (e.g. Raa Atoll, No. 15; reported in official census statistics, see^30^). It may be vegetated or not and, in the first case, its vegetation may be either native, or introduced (e.g. coconut groves), or even mixed.
- ‘Island land area’ corresponds to the stabilised part of an island, calculated using the vegetation line as a shoreline proxy, as in most atoll studies (e.g.^19^).
- An ‘exploited’ island is an island that is dedicated to specific economic activities and/or infrastructures, including tourism (resort island), industry, transport facilities (airport), agriculture, aquaculture, etc. Islands exhibiting unexploited coconut groves are not included in this category, but considered as ‘natural’.
- ‘Land reclamation’ corresponds to artificial land area gain through the landfill of one or more reef flat area(s). Reclaimed areas are included in island land area estimates.
- ‘Shoreline armouring’ or ‘hardening’ indicates the replacement of natural shoreline by artificial shoreline due to land reclamation or to the construction of engineered structures, such as coastal protection structures (e.g. seawalls, riprap, etc.) or structures associated with coastal development (e.g. harbour jetties). Fixed shoreline corresponds to the proportion of island shoreline that has been armoured.
- A ‘proper harbour’ designates a harbour having engineered structures, i.e. either an alongshore or a transversal quay (e.g. Haa Alifu-Noonu No. 135; Lhaviyani No, 12), or protective structures, such as foreshore breakwaters. ‘Proper harbours’ may therefore either be fully open to the open sea, or ‘closed’ by a jetty or breakwater (e.g. Lhaviyani No5), and may include or not a dredged basin (e.g. Gaafu Alifu-Dhaalu No110). They show various configurations, from ‘traditional’ to ‘modern’. We excluded small mooring areas exhibiting no engineered structures (e.g. Ihavandhippolhu, islands Nos 2 and 11, ocean coast). Harbour extensions are included in shoreline estimates.
  Harbour inventory excludes harbours that are no longer functional (e.g. Haa Alifu-Noonu Nos 21-22). Nearly-completed harbours are included (e.g. Haa Alifu-Noonu No. 85).
- ‘Harbour basins dredged in reef flat include the basins of both ‘proper harbours’ and harbours having no stabilised shoreline or quay.
- Wharves are mainly present on inhabited and resort islands. They do not include the pontoons connecting water bungalows to the coast on resort islands. They may intercept the longshore sediment drift or not, depending on their design (permeable or not).
- ‘Sediment dredging from reef flat’ excludes dredging works related to the establishment of boat channels and harbours, which are covered by dedicated variables.

### Inventory and classification of human disturbances affecting the ‘reef-island system’

The ‘reef-island system’ considered in this study (Fig. 1) includes two interdependent geomorphic features, that is, the island and the reef ecosystem that supports it, which supplies the island with sediment and attenuates wave energy, especially generated by storms. Human disturbances affecting the reef-island system were assessed based on satellite image analysis, using high resolution (0.50 m) images from two time periods, 2004–06 and 2014–16, and freely provided by Google Earth. Such free remote-sensing data were already used to conduct nation-wide assessments, e.g. to assess land use change in the Maldives^35^. Image interpretation benefited from the conduction by the authors of fieldwork on some atolls, namely North Kaafu, South Kaafu and Seenu.

The human disturbances considered in this study, which are as follows, were classified into three categories, depending on the part of the reef-island system that they affect:

1. *The degree of human occupancy of each island* was assessed using the ‘Human Footprint’ (see definition above) as a proxy.
2. *The human pressure exerted on the coast* was assessed based on the inventory of human activities that disrupt coastal dynamics, including coastal developments, infrastructures and human-built structures. The latter include reclaimed plots protruding onto the reef flat, harbours, longitudinal (mostly seawalls and riprap) and transversal (i.e. groynes) coastal protection structures, and marine (mostly breakwaters) protection structures. Beyond documenting the occurrence of and change in the extent of such disturbances, we also documented Shoreline (S) type (classified into 5 classes, as follows: “entirely natural”, S1; “predominantly natural”, S2; and “half-natural, half-fixed”, “predominantly fixed”, and “entirely fixed” by human-built structures, respectively S3, S4 and S5), using it as a proxy to quantify the degree of disturbance of island shoreline.
3. *The human pressure exerted on the island’s reef* was assessed based on the inventory of harbour basins dredged in, sediment mining from, and boat channels dredged across the island’s reef flat. Beyond documenting the occurrence of and change in the extent of such human disturbances, we aggregated these variables into a synthetic index (“human pressure exerted on island Reef”, called R for “reef”) that allowed classifying islands into three distinct categories:

- R1 corresponds to islands exhibiting *no-to-limited human modification of the island’s reef*. These islands exhibit no direct (water pollution is excluded due to data unavailability) and visually detectable (i.e. on the satellite images used) human disturbance. This means that some disturbances (e.g. aggregate extraction) may occur that are not considered in this study because they were not detectable on the satellite images used.
- R2 corresponds to islands exhibiting *moderate human pressure on the island’s reef*. “Moderate” means that these islands have ≤1 harbour basin dredged in the reef flat, and may also exhibit other disturbances, such as boat channels dredged across and/or sediment dredging from the island’s reef flat and/or marine protection structures.
- R3 corresponds to islands exhibiting *high to very high human pressure on the island’s reef*. “High to very high” means that these islands have more than one harbour basin dredged in the reef flat (i.e. 2 or more harbour basins), and may also exhibit other disturbances, such as boat channels dredged across and/or sediment dredging from the island’s reef flat and/or marine protection structures. Because they have several harbours dredged in the reef flat, generally on both their lagoon and ocean sides, these islands exhibit highly to very highly disturbed sediment production and transport patterns.

### Data generation and analysis

The only official (i.e. established by the Government of the Maldives) data used in this study relates to population number, for which we used national censuses (Statistical Yearbooks 2006, 2016 and 2017; available here: http://statisticsmaldives.gov.mv/yearbook/). It is noteworthy that there is no official dataset providing comprehensive and reliable information on the other variables considered in this study. In some cases, some data are available (e.g. for island land area), but because these data are provided without their metadata (i.e. no information on how and when they were generated), we decided to not use them. Moreover, for those variables for which official data exist, one single dataset was generally available, which did not allow the analysis of change. For other variables (e.g. land reclamation), existing datasets are incomplete, that is, not updated, and not always covering all of the Maldives Islands. Due to these constraints, we generated all of the data that are used in this study, using high resolution (0.5 m) and good quality (i.e. excluding images exhibiting cloud cover) satellite images from the 2004–06 and 2014–16 periods that are freely provided by Google Earth. Due to image availability constraint, we used a set of images (and therefore different images from one island to another) to document the situation of each island over the two periods considered in this study. The precise date of the images used for each island is indicated in the database delivered with this paper. Data were generated based on satellite image interpretation, in line with previous studies on atoll island and shoreline change^19,23–25,27,28^. However, this study includes the assessment of variables that were never assessed in previous studies, for which the methods used are presented hereinafter. The full database is presented in Supplementary Material S6 (xls file) and S7 (kmz file).

### Human footprint (HF)

For each island, we derived the HF from image analysis and classified the spatial extent of HF into 4 categories: HF = 0 (corresponds to ‘natural’ islands); HF ≤ 1/3, 1/3 < HF ≤ 2/3, HF > 2/3. Changes were assessed based on these four categories, which allowed highlighting changes from ‘natural’ to ‘exploited’ islands, and changes in island category over the period of analysis.

### Land reclamation

Land reclamation was assessed based on image interpretation, similar to previous atoll studies^24,25,27–29^. On atoll islands, artificial island areas resulting from landfill are generally easily detectable on satellite images, as they are indicated by the geometric shape of concerned island areas and related shoreline, and by the absence, limited coverage or different type of vegetation in these areas compared to the rest of the island. As reclaimed areas are generally made of reef sediments that are directly dredged from the island’s reef flat, land reclamation is generally also indicated by extensive sediment dredging from the reef flat.

### Longitudinal and transversal coastal protection structures

‘Coastal protection structures’ include all of the protection structures that were erected along the shoreline, including the ocean, lagoon and *hoa* (i.e. inter-islet channel) shoreline, and the shoreline of inner lagoons (e.g. Haa Alifu-Noonu Atoll, No96). The structures that were erected on the reef flat (e.g. foreshore breakwaters) are included in the marine protection structures category. ‘Coastal protection structures’ may be either proper engineered structures (i.e. well-designed and calibrated structures, e.g. along reclaimed areas), or handmade poorly designed and built structures. Both types are included in this assessment, with no distinction made between them.

‘Longitudinal coastal structures’ include not only the structures that were built along the shoreline to fix it, but also the structures (generally riprap) that were erected to stabilise reclaimed areas (e.g. Haa Alifu-Noonu Atoll, No5). Of note, some reclaimed areas are not stabilised by protection structures. Stabilised reclaimed areas are common on both sides of harbours, as the dredging of harbour basins in reef flat provides materials that are commonly used to reclaim nearby areas.

The establishment of additional structures between the first and the second date was assessed based on a binary basis (yes = 1/no = 0).

The limited vegetation cover occurring along the shoreline of most islands (as a result of vegetation clearing or entire removal), facilitated the detection of longitudinal coastal structures, which mainly consist of seawalls and riprap^39,40^. However, 43 occurrences (distributed among 31 islands) out of 536 (8%) were uncertain, due to the localised masking of the shoreline (either by cloud cover, or by the coastal vegetation) and to the nature of structures (i.e. small, or made of hardly detectable materials). This limitation does not concern transversal coastal structures (i.e. groynes) and marine protection structures (consisting of foreshore and nearshore breakwaters) that were easily detected on the images used.

### Shoreline type (S)

Shoreline type refers to natural vs. fixed (i.e. by human-built structures) shoreline. Fixed shoreline includes the shoreline of reclaimed plots that were stabilised by engineered structures (mainly riprap).

### Island land area

Island land area was calculated at each date, using the stability line as a shoreline proxy, as in previous atoll studies^29,44^. The stability line corresponds to the vegetation line on natural shoreline and to the seaward limit of human-built structures on artificial coastline. In line with previous studies, we used the ±3% threshold to determine significant change, considering that a change falling within –3% and +3% was not significant, that is, indicated areal stability, while changes <–3% and >3% respectively corresponded to island contraction and expansion. We classified island areal growth into 5 distinct categories (3 ≤ x < 10%; 10 ≤ x < 25%; 25 ≤ x < 50%; 50 ≤ x < 100%; x ≥ 100%) in order to highlight the contrasting situations of the sample islands.

### Harbour basin and boat channel dredged in reef flat, and sediment dredging from reef flat

Human activities involving dredging in reef flat are generally detectable on satellite images. Dredging is indicated by a change in the nature (i.e. colour and texture) of the reef flat and by the geometric contours of dredged areas.

For a proper harbour having a basin dredged in reef flat, both the proper harbour and the basin dredged in reef flat were counted, so as to allow appreciating the modifications caused both to the coast (i.e. artificial shoreline and changes in currents and associated sediment transport) and to the reef (degradation of the reef ecosystem). Of note, when a harbour or a harbour basin was situated between two islands located on the same reef flat, it was counted for each island, i.e. double counted (e.g. Seenu, Nos11 and 13).

Data processing and cross-analysis was conducted using Excel software, and allowed generating the results that are presented in the Supplementary Materials.

### Determination of island Types (T)

We aggregated Shoreline (S) and Reef (R) indicators to generate 5 island types reflecting the degree of human disturbance of the reef-island system. The 5 island types were established as follows:

T1 (island Type 1) – No human disturbance: S1 + R1

T2 (island Type 2) – Low human disturbance: S1 + R2, S2 + R1

T3 (island Type 3) – Moderate human disturbance: S1 + R3, S2 + R2, S2 + R3

T4 (island Type 4) – High human disturbance: S3 + R1, S3 + R2, S3 + R3

T5 (island Type 5) – Very high human disturbance: S4 + R1, S4 + R2, S4 + R3, S5 + R1, S5 + R2, S5 + R3

## Supplementary information

Supplementary Information S1-S5

Supplementary Information S6

Supplementary Information S7

## Data Availability

All data generated during this study are included in this published article (and its Supplementary Materials files).
