# Publisher Correction: Rapid human-driven undermining of atoll island capacity to adjust to ocean climate-related pressures

**DOI:** 10.1038/s41598-019-54659-0

**Published:** 2019-11-29

**Authors:** Virginie K. E. Duvat, Alexandre K. Magnan

**Affiliations:** 1 UMR LIENSs 7266, La Rochelle University-CNRS – 2 rue Olympe de Gouges, 17000 La Rochelle, France; 2 Institute for Sustainable Development and International Relations – Sciences Po, 27 rue Saint Guillaume, 75007 Paris, France

Correction to: *Scientific Reports* 10.1038/s41598-019-51468-3, published online 22 October 2019

In the Supplementary Information file originally published with this Article, the wrong version of Supplementary Information file S1–S5 was published. This has now been corrected in the Supplementary Information that now accompanies the Article.

