# Supplementary material for: Rapid human-driven undermining of atoll island capacity to adjust to ocean climate-related pressures
Source: Sci Rep. 2019 Oct 22;9:15129. doi: 10.1038/s41598-019-51468-3 (PMC6805953; doi:10.1038/s41598-019-51468-3)
Supplement: Supplementary file 1 — Supplementary Information S1-S5 [file 41598_2019_51468_MOESM1_ESM.pdf]

S1 – Change in Human Footprint (HF) between 2004-2006 and 2014-2016

Content

Table A. Change in Human Footprint (HF, per atoll)
Table B. Change in Human Footprint (HF, all atolls)

Table A. Change in Human Footprint (HF, per atoll)

1. Ihavandhippolhu

		2014-2016						2014-2016				
		Number of islands						% of the atoll sample				
		HF = 0	HF ≤1/3	1/3 < HF ≤ 2/3	HF > 2/3	Total		HF = 0	HF ≤ 1/3	1/3 < HF ≤ 2/3	HF > 2/3	Total
2004-2006	HF = 0	10	0	0	1	11	HF = 0	55.6	0.0	0.0	5.6	61.1
	HF < 1/3	0	2	1	0	3	HF < 1/3	0.0	11.1	5.6	0.0	16.7
	1/3 < HF < 2/3	0	0	0	1	1	1/3 < HF < 2/3	0.0	0.0	0.0	5.6	5.6
	HF > 2/3	0	0	0	3	3	HF > 2/3	0.0	0.0	0.0	16.7	16.7
	Total	10	2	1	5	18	Total	55.6	11.1	5.6	27.8	100.0

2. Haa Alifu-Noonu

		2014-2016						2014-2016				
		Number of islands						% of the atoll sample				
		HF = 0	HF ≤ 1/3	1/3 < HF ≤2/3	HF > 2/3	Total		HF = 0	HF ≤ 1/3	1/3 < HF ≤ 2/3	HF > 2/3	Total
2004-2006	HF = 0	19	3	0	1	23	HF = 0	32.8	5.2	0.0	1.7	39.7
	HF < 1/3	1	4	7	2	14	HF < 1/3	1.7	6.9	12.1	3.4	24.1
	1/3 < HF < 2/3	2	0	3	5	10	1/3 < HF < 2/3	3.4	0.0	5.2	8.6	17.2
	HF > 2/3	0	0	0	11	11	HF > 2/3	0.0	0.0	0.0	19.0	19.0
	Total	22	7	10	19	58	Total	37.9	12.1	17.2	32.8	100.0

3. Maamakunudhoo

		2014-2016 Number of islands						2014-2016 % of the atoll sample				
		HF = 0	HF ≤ 1/3	1/3 < HF ≤ 2/3	HF > 2/3	Total		HF = 0	HF ≤ 1/3	1/3 < HF ≤ 2/3	HF > 2/3	Total
2004-2006	HF = 0	1	0	0	0	1	HF = 0	50.0	0.0	0.0	0.0	50.0
	HF < 1/3	0	0	0	0	0	HF < 1/3	0.0	0.0	0.0	0.0	0.0
	1/3 < HF < 2/3	0	0	0	1	1	1/3 < HF < 2/3	0.0	0.0	0.0	50.0	50.0
	HF > 2/3	0	0	0	0	0	HF > 2/3	0.0	0.0	0.0	0.0	0.0
	Total	1	0	0	1	2	Total	50.0	0.0	0.0	50.0	100.0

4. Raa

		2014-2016 Number of islands						2014-2016 % of the atoll sample				
		HF = 0	HF ≤ 1/3	1/3 < HF ≤ 2/3	HF > 2/3	Total		HF = 0	HF ≤ 1/3	1/3 < HF ≤ 2/3	HF > 2/3	Total
2004-2006	HF = 0	24	5	1	4	34	HF = 0	50.0	10.4	2.1	8.3	70.8
	HF < 1/3	0	0	2	2	4	HF < 1/3	0.0	0.0	4.2	4.2	8.3
	1/3 < HF < 2/3	0	0	1	1	2	1/3 < HF < 2/3	0.0	0.0	2.1	2.1	4.2
	HF > 2/3	1	0	0	7	8	HF > 2/3	2.1	0.0	0.0	14.6	16.7
	Total	25	5	4	14	48	Total	52.1	10.4	8.3	29.2	100.0

5. Lhaviyani

		2014-2016 Number of islands						2014-2016 % of the atoll sample				
		HF = 0	HF ≤ 1/3	1/3 < HF ≤ 2/3	HF > 2/3	Total		HF = 0	HF ≤ 1/3	1/3 < HF ≤ 2/3	HF > 2/3	Total
2004-2006	HF = 0	12	1	1	3	17	HF = 0	46.2	3.8	3.8	11.5	65.4
	HF < 1/3	0	1	0	0	1	HF < 1/3	0.0	3.8	0.0	0.0	3.8
	1/3 < HF < 2/3	0	0	1	0	1	1/3 < HF < 2/3	0.0	0.0	3.8	0.0	3.8
	HF > 2/3	0	0	0	7	7	HF > 2/3	0.0	0.0	0.0	26.9	26.9
	Total	12	2	2	10	26	Total	46.2	7.7	7.7	38.5	100.0

6. Baa

		2014-2016 Number of islands						2014-2016 % of the atoll sample				
		HF = 0	HF ≤ 1/3	1/3 < HF ≤ 2/3	HF > 2/3	Total		HF = 0	HF ≤ 1/3	1/3 < HF ≤ 2/3	HF > 2/3	Total
2004-2006	HF = 0	20	3	1	3	27	HF = 0	45.5	6.8	2.3	6.8	61.4
	HF < 1/3	0	3	1	1	5	HF < 1/3	0.0	6.8	2.3	2.3	11.4
	1/3 < HF < 2/3	0	0	1	1	2	1/3 < HF < 2/3	0.0	0.0	2.3	2.3	4.5
	HF > 2/3	0	0	0	10	10	HF > 2/3	0.0	0.0	0.0	22.7	22.7
	Total	20	6	3	15	44	Total	45.5	13.6	6.8	34.1	100.0

7. Kaashidhoo

		2014-2016 Number of islands						2014-2016 % of the atoll sample				
		HF = 0	HF ≤ 1/3	1/3 < HF ≤ 2/3	HF > 2/3	Total		HF = 0	HF ≤ 1/3	1/3 < HF ≤ 2/3	HF > 2/3	Total
2004-2006	HF = 0	0	0	0	0	0	HF = 0	0.0	0.0	0.0	0.0	0.0
	HF < 1/3	0	0	0	0	0	HF < 1/3	0.0	0.0	0.0	0.0	0.0
	1/3 < HF < 2/3	0	0	0	1	1	1/3 < HF < 2/3	0.0	100.0	0.0	0.0	100.0
	HF > 2/3	0	0	0	0	0	HF > 2/3	0.0	0.0	0.0	0.0	0.0
	Total	0	0	0	1	1	Total	0.0	100.0	0.0	0.0	100.0

8. Goidhoo

		2014-2016 Number of islands						2014-2016 % of the atoll sample				
		HF = 0	HF ≤ 1/3	1/3 < HF ≤ 2/3	HF > 2/3	Total		HF = 0	HF ≤ 1/3	1/3 < HF ≤ 2/3	HF > 2/3	Total
2004-2006	HF = 0	1	0	0	0	1	HF = 0	100.0	0.0	0.0	0.0	100.0
	HF < 1/3	0	0	0	0	0	HF < 1/3	0.0	0.0	0.0	0.0	0.0
	1/3 < HF < 2/3	0	0	0	0	0	1/3 < HF < 2/3	0.0	0.0	0.0	0.0	0.0
	HF > 2/3	0	0	0	0	0	HF > 2/3	0.0	0.0	0.0	0.0	0.0
	Total	1	0	0	0	1	Total	100.0	0.0	0.0	0.0	100.0

9. Gaafaru

		2014-2016 Number of islands						2014-2016 % of the atoll sample				
		HF = 0	HF ≤ 1/3	1/3 < HF ≤ 2/3	HF > 2/3	Total		HF = 0	HF ≤ 1/3	1/3 < HF ≤ 2/3	HF > 2/3	Total
2004-2006	HF = 0	0	0	0	0	0	HF = 0	0.0	0.0	0.0	0.0	0.0
	HF < 1/3	0	0	0	0	0	HF < 1/3	0.0	0.0	0.0	0.0	0.0
	1/3 < HF < 2/3	0	0	0	0	0	1/3 < HF < 2/3	0.0	0.0	0.0	0.0	0.0
	HF > 2/3	0	0	0	1	1	HF > 2/3	0.0	0.0	0.0	100.0	100.0
	Total	0	0	0	1	1	Total	0.0	0.0	0.0	100.0	100.0

10. North Kaafu

		2014-2016 Number of islands						2014-2016 % of the atoll sample				
		HF = 0	HF ≤ 1/3	1/3 < HF ≤ 2/3	HF > 2/3	Total		HF = 0	HF ≤ 1/3	1/3 < HF ≤ 2/3	HF > 2/3	Total
2004-2006	HF = 0	0	3	0	2	5	HF = 0	0.0	7.5	0.0	5.0	12.5
	HF < 1/3	0	1	0	0	1	HF < 1/3	0.0	2.5	0.0	0.0	2.5
	1/3 < HF < 2/3	0	0	1	1	2	1/3 < HF < 2/3	0.0	0.0	2.5	2.5	5.0
	HF > 2/3	0	0	1	31	32	HF > 2/3	0.0	0.0	2.5	77.5	80.0
	Total	0	4	2	34	40	Total	0.0	10.0	5.0	85.0	100.0

11. Thoddoo

		2014-2016 Number of islands						2014-2016 % of the atoll sample				
		HF = 0	HF ≤ 1/3	1/3 < HF ≤ 2/3	HF > 2/3	Total		HF = 0	HF ≤ 1/3	1/3 < HF ≤ 2/3	HF > 2/3	Total
2004-2006	HF = 0	0	0	0	0	0	HF = 0	0.0	0.0	0.0	0.0	0.0
	HF < 1/3	0	0	0	0	0	HF < 1/3	0.0	0.0	0.0	0.0	0.0
	1/3 < HF < 2/3	0	0	0	0	0	1/3 < HF < 2/3	0.0	0.0	0.0	0.0	0.0
	HF > 2/3	0	0	0	1	1	HF > 2/3	0.0	0.0	0.0	100.0	100.0
	Total	0	0	0	1	1	Total	0.0	0.0	0.0	100.0	100.0

12. Rasdhoo

		2014-2016 Number of islands						2014-2016 % of the atoll sample				
		HF = 0	HF ≤ 1/3	1/3 < HF ≤ 2/3	HF > 2/3	Total		HF = 0	HF ≤ 1/3	1/3 < HF ≤ 2/3	HF > 2/3	Total
2004-2006	HF = 0	1	0	0	0	1	HF = 0	16.7	0.0	0.0	0.0	16.7
	HF < 1/3	0	1	0	0	1	HF < 1/3	0.0	16.7	0.0	0.0	16.7
	1/3 < HF < 2/3	0	0	0	0	0	1/3 < HF < 2/3	0.0	0.0	0.0	0.0	0.0
	HF > 2/3	0	0	0	4	4	HF > 2/3	0.0	0.0	0.0	66.7	66.7
	Total	1	1	0	4	6	Total	16.7	16.7	0.0	66.7	100.0

13. Alifu

		2014-2016 Number of islands						2014-2016 % of the atoll sample				
		HF = 0	HF ≤ 1/3	1/3 < HF ≤ 2/3	HF > 2/3	Total		HF = 0	HF ≤ 1/3	1/3 < HF ≤ 2/3	HF > 2/3	Total
2004-2006	HF = 0	2	0	0	0	2	HF = 0	11.1	0.0	0.0	0.0	11.1
	HF < 1/3	0	1	0	0	1	HF < 1/3	0.0	5.6	0.0	0.0	5.6
	1/3 < HF < 2/3	0	1	0	3	4	1/3 < HF < 2/3	0.0	5.6	0.0	16.7	22.2
	HF > 2/3	0	0	0	11	11	HF > 2/3	0.0	0.0	0.0	61.1	61.1
	Total	2	2	0	14	18	Total	11.1	11.1	0.0	77.8	100.0

14. South Kaafu

		2014-2016 Number of islands						2014-2016 % of the atoll sample				
		HF = 0	HF ≤ 1/3	1/3 < HF ≤ 2/3	HF > 2/3	Total		HF = 0	HF ≤ 1/3	1/3 < HF ≤ 2/3	HF > 2/3	Total
2004-2006	HF = 0	2	0	0	0	2	HF = 0	8.3	0.0	0.0	0.0	8.3
	HF < 1/3	0	1	0	0	1	HF < 1/3	0.0	4.2	0.0	0.0	4.2
	1/3 < HF < 2/3	0	0	0	1	1	1/3 < HF < 2/3	0.0	0.0	0.0	4.2	4.2
	HF > 2/3	0	0	0	20	20	HF > 2/3	0.0	0.0	0.0	83.3	83.3
	Total	2	1	0	21	24	Total	8.3	4.2	0.0	87.5	100.0

15. Vaavu

		2014-2016 Number of islands						2014-2016 % of the atoll sample				
		HF = 0	HF ≤ 1/3	1/3 < HF ≤ 2/3	HF > 2/3	Total		HF = 0	HF ≤ 1/3	1/3 < HF ≤ 2/3	HF > 2/3	Total
2004-2006	HF = 0	1	0	0	0	1	HF = 0	14.3	0.0	0.0	0.0	14.3
	HF < 1/3	0	1	0	1	2	HF < 1/3	0.0	14.3	0.0	14.3	28.6
	1/3 < HF < 2/3	0	0	0	0	0	1/3 < HF < 2/3	0.0	0.0	0.0	0.0	0.0
	HF > 2/3	0	0	0	4	4	HF > 2/3	0.0	0.0	0.0	57.1	57.1
	Total	1	1	0	5	7	Total	14.3	14.3	0.0	71.4	100.0

16. Faafu

		2014-2016 Number of islands						2014-2016 % of the atoll sample				
		HF = 0	HF ≤ 1/3	1/3 < HF ≤ 2/3	HF > 2/3	Total		HF = 0	HF ≤ 1/3	1/3 < HF ≤ 2/3	HF > 2/3	Total
2004-2006	HF = 0	4	0	0	0	4	HF = 0	50.0	0.0	0.0	0.0	50.0
	HF < 1/3	0	0	0	0	0	HF < 1/3	0.0	0.0	0.0	0.0	0.0
	1/3 < HF < 2/3	0	0	1	1	2	1/3 < HF < 2/3	0.0	0.0	12.5	12.5	25.0
	HF > 2/3	0	0	0	2	2	HF > 2/3	0.0	0.0	0.0	25.0	25.0
	Total	4	0	1	3	8	Total	50.0	0.0	12.5	37.5	100.0

17. Meemu

		2014-2016						2014-2016				
		Number of islands						% of the atoll sample				
		HF = 0	HF ≤ 1/3	1/3 < HF ≤ 2/3	HF > 2/3	Total		HF = 0	HF ≤ 1/3	1/3 < HF ≤ 2/3	HF > 2/3	Total
2004-2006	HF = 0	12	2	1	0	15	HF = 0	54.5	9.1	4.5	0.0	68.2
	HF < 1/3	0	1	0	0	1	HF < 1/3	0.0	4.5	0.0	0.0	4.5
	1/3 < HF < 2/3	0	0	1	1	2	1/3 < HF < 2/3	0.0	0.0	4.5	4.5	9.1
	HF > 2/3	0	0	0	4	4	HF > 2/3	0.0	0.0	0.0	18.2	18.2
	Total	12	3	2	5	22	Total	54.5	13.6	9.1	22.7	100.0

18. Dhaalu

		2014-2016						2014-2016				
		Number of islands						% of the atoll sample				
		HF = 0	HF ≤ 1/3	1/3 < HF ≤ 2/3	HF > 2/3	Total		HF = 0	HF ≤ 1/3	1/3 < HF ≤ 2/3	HF > 2/3	Total
2004-2006	HF = 0	2	0	0	3	5	HF = 0	22.2	0.0	0.0	33.3	55.6
	HF < 1/3	0	0	0	0	0	HF < 1/3	0.0	0.0	0.0	0.0	0.0
	1/3 < HF < 2/3	0	0	0	0	0	1/3 < HF < 2/3	0.0	0.0	0.0	0.0	0.0
	HF > 2/3	0	0	0	4	4	HF > 2/3	0.0	0.0	0.0	44.4	44.4
	Total	2	0	0	7	9	Total	22.2	0.0	0.0	77.8	100.0

19. Thaa

		2014-2016						2014-2016				
		Number of islands						% of the atoll sample				
		HF = 0	HF ≤ 1/3	1/3 < HF ≤ 2/3	HF > 2/3	Total		HF = 0	HF ≤ 1/3	1/3 < HF ≤ 2/3	HF > 2/3	Total
2004-2006	HF = 0	17	0	0	0	17	HF = 0	73.9	0.0	0.0	0.0	73.9
	HF < 1/3	0	0	0	0	0	HF < 1/3	0.0	0.0	0.0	0.0	0.0
	1/3 < HF < 2/3	0	0	0	2	2	1/3 < HF < 2/3	0.0	0.0	0.0	8.7	8.7
	HF > 2/3	0	0	0	4	4	HF > 2/3	0.0	0.0	0.0	17.4	17.4
	Total	17	0	0	6	23	Total	73.9	0.0	0.0	26.1	100.0

20. Laamu

		2014-2016 Number of islands						2014-2016 % of the atoll sample				
		HF = 0	HF ≤ 1/3	1/3 < HF ≤ 2/3	HF > 2/3	Total		HF = 0	HF ≤ 1/3	1/3 < HF ≤ 2/3	HF > 2/3	Total
2004-2006	HF = 0	21	2	1	0	24	HF = 0	56.8	5.4	2.7	0.0	64.9
	HF < 1/3	1	3	1	0	5	HF < 1/3	2.7	8.1	2.7	0.0	13.5
	1/3 < HF < 2/3	0	0	2	1	3	1/3 < HF < 2/3	0.0	0.0	5.4	2.7	8.1
	HF > 2/3	1	0	0	4	5	HF > 2/3	2.7	0.0	0.0	10.8	13.5
	Total	23	5	4	5	37	Total	62.2	13.5	10.8	13.5	100.0

21. Gaafu Alifu-Dhaalu

		2014-2016 Number of islands						2014-2016 % of the atoll sample				
		HF = 0	HF ≤ 1/3	1/3 < HF ≤ 2/3	HF > 2/3	Total		HF = 0	HF ≤ 1/3	1/3 < HF ≤ 2/3	HF > 2/3	Total
2004-2006	HF = 0	151	4	3	6	164	HF = 0	81.2	2.2	1.6	3.2	88.2
	HF < 1/3	2	5	0	2	9	HF < 1/3	1.1	2.7	0.0	1.1	4.8
	1/3 < HF < 2/3	0	0	1	5	6	1/3 < HF < 2/3	0.0	0.0	0.5	2.7	3.2
	HF > 2/3	0	0	0	7	7	HF > 2/3	0.0	0.0	0.0	3.8	3.8
	Total	153	9	4	20	186	Total	82.3	4.8	2.2	10.8	100.0

22. Gnaviyani

		2014-2016 Number of islands						2014-2016 % of the atoll sample				
		HF = 0	HF ≤ 1/3	1/3 < HF ≤ 2/3	HF > 2/3	Total		HF = 0	HF ≤ 1/3	1/3 < HF ≤ 2/3	HF > 2/3	Total
2004-2006	HF = 0	0	0	0	0	0	HF = 0	0.0	0.0	0.0	0.0	0.0
	HF < 1/3	0	0	0	0	0	HF < 1/3	0.0	0.0	0.0	0.0	0.0
	1/3 < HF < 2/3	0	0	0	0	0	1/3 < HF < 2/3	0.0	0.0	0.0	0.0	0.0
	HF > 2/3	0	0	0	1	1	HF > 2/3	0.0	0.0	0.0	100.0	100.0
	Total	0	0	0	1	1	Total	0.0	0.0	0.0	100.0	100.0

23. Seenu

		2014-2016 Number of islands						2014-2016 % of the atoll sample				
		HF = 0	HF ≤ 1/3	1/3 < HF ≤ 2/3	HF > 2/3	Total		HF = 0	HF ≤ 1/3	1/3 < HF ≤ 2/3	HF > 2/3	Total
2004-2006	HF = 0	18	1	0	1	20	HF = 0	66.7	3.7	0.0	3.7	74.1
	HF < 1/3	1	0	0	1	2	HF < 1/3	3.7	0.0	0.0	3.7	7.4
	1/3 < HF < 2/3	0	0	0	1	1	1/3 < HF < 2/3	0.0	0.0	0.0	3.7	3.7
	HF > 2/3	0	0	0	4	4	HF > 2/3	0.0	0.0	0.0	14.8	14.8
	Total	19	1	0	7	27	Total	70.4	3.7	0.0	25.9	100.0

Table B. Change in Human Footprint (HF, all atolls)

		2014-2016				
		**Number of islands**				
		**HF = 0**	**HF ≤ 1/3**	**1/3 < HF ≤ 2/3**	**HF > 2/3**	**Total**
**2004-2006**	**HF = 0**	318	24	8	24	374
	**HF < 1/3**	5	24	12	9	50
	**1/3 < HF < 2/3**	2	1	12	26	41
	**HF > 2/3**	2	0	1	140	143
	**Total**	327	49	33	199	608

		2014-2016				
		**% of the atoll sample**				
		**HF = 0**	**HF ≤ 1/3**	**1/3 < HF ≤ 2/3**	**HF > 2/3**	**Total**
**2004-2006**	**HF = 0**	52.3	3.9	1.3	3.9	61.5
	**HF < 1/3**	0.8	3.9	2.0	1.5	8.2
	**1/3 < HF < 2/3**	0.3	0.2	2.0	4.3	6.7
	**HF > 2/3**	0.3	0.0	0.2	23.0	23.5
	**Total**	53.8	8.1	5.4	32.7	100.0

**Nb**	**%**	**Change in HF**
11	1.8	Decrease
494	81.3	Stability
103	16.9	Increase
608	100.0	Total

S2 – Human-driven coastal change between 2004-2006 and 2014-2016

<u>Content</u>

Table A. Change in coastal developments and infrastructures
Table B. Change in defence structures
Table C. Change in shoreline type (per atoll)
Table D. Change in shoreline type (all atolls)

Table A. Change in coastal developments and infrastructures

	Nb of islands		% (in proportion to the number of islands per atoll)		Rate of change in the number of equipped islands (+ X%), in proportion to the number of islands per atoll	Rate of change in the % of equipped islands (+ X%), in proportion to the number of islands per atoll
	2004-2006	2014-2016	2004-2006	2014-2016		
**Ihavandhippolhu**						
Land reclamation	2	5	11.1	27.8	150.0	16.7
Proper harbour	2	5	11.1	27.8	150.0	16.7
Wharf	3	2	16.7	11.1	-33.3	-5.6
**Total atoll sample**	**18**					
**Haa Alifu-Noonu**						
Land reclamation	8	24	13.8	41.4	200.0	27.6
Proper harbour	7	24	12.1	41.4	242.9	29.3
Wharf	12	15	20.7	25.9	25.0	5.2
**Total atoll sample**	**58**					
**Maamakunudhoo**						
Land reclamation	0	1	0.0	50.0	100.0	50.0
Proper harbour	1	1	50.0	50.0	0.0	0.0
Wharf	0	0	0.0	0.0	100.0	0.0
**Total atoll sample**	**2**					
**Raa**						
Land reclamation	6	14	12.5	29.2	133.3	16.7
Proper harbour	6	13	12.5	27.1	116.7	14.6
Wharf	3	7	6.3	14.6	133.3	8.3
**Total atoll sample**	**48**					
**Lhaviyani**						
Land reclamation	5	9	19.2	34.6	80.0	15.4
Proper harbour	5	6	19.2	23.1	20.0	3.8
Wharf	4	6	15.4	23.1	50.0	7.7
**Total atoll sample**	**26**					
**Baa**						
Land reclamation	8	10	18.2	22.7	25.0	4.5
Proper harbour	7	9	15.9	20.5	28.6	4.5
Wharf	5	11	11.4	25.0	120.0	13.6
**Total atoll sample**	**44**					
**Kaashidhoo**						
Land reclamation	1	1	100.0	100.0	0.0	0.0
Proper harbour	1	1	100.0	100.0	0.0	0.0
Wharf	0	0	0.0	0.0	0.0	0.0
**Total atoll sample**	**1**					

**Goidhoo**						
Land reclamation	0	0	0.0	0.0	/	0.0
Proper harbour	0	0	0.0	0.0	/	0.0
Wharf	0	0	0.0	0.0	0.0	0.0
**Total atoll sample**	**1**					
**Gaafaru**						
Land reclamation	1	1	100.0	100.0	0.0	0.0
Proper harbour	1	1	100.0	100.0	0.0	0.0
Wharf	0	0	0.0	0.0	0.0	0.0
**Total atoll sample**	**1**					
**North Kaafu**						
Land reclamation	28	30	70.0	75.0	7.1	5.0
Proper harbour	20	21	50.0	52.5	5.0	2.5
Wharf	14	15	35.0	37.5	7.1	2.5
**Total atoll sample**	**40**					
**Thoddoo**						
Land reclamation	1	1	100.0	100.0	0.0	0.0
Proper harbour	1	1	100.0	100.0	0.0	0.0
Wharf	0	0	0.0	0.0	0.0	0.0
**Total atoll sample**	**1**					
**Rasdhoo**						
Land reclamation	2	2	33.3	33.3	0.0	0.0
Proper harbour	1	2	16.7	33.3	100.0	16.7
Wharf	3	2	50.0	33.3	-33.3	-16.7
**Total atoll sample**	**6**					
**Alifu**						
Land reclamation	7	8	38.9	44.4	14.3	5.6
Proper harbour	5	6	27.8	33.3	20.0	5.6
Wharf	9	8	50.0	44.4	-11.1	-5.6
**Total atoll sample**	**18**					
**South Kaafu**						
Land reclamation	13	16	54.2	66.7	23.1	12.5
Proper harbour	7	10	29.2	41.7	42.9	12.5
Wharf	13	11	54.2	45.8	-15.4	-8.3
**Total atoll sample**	**24**					
**Vaavu**						
Land reclamation	3	5	42.9	71.4	66.7	28.6
Proper harbour	1	3	14.3	42.9	200.0	28.6
Wharf	4	3	57.1	42.9	-25.0	-14.3
**Total atoll sample**	**7**					
**Faafu**						
Land reclamation	2	3	25.0	37.5	50.0	12.5
Proper harbour	1	2	12.5	25.0	100.0	12.5
Wharf	3	3	37.5	37.5	0.0	0.0
**Total atoll sample**	**8**					
**Meemu**						
Land reclamation	3	5	13.6	22.7	66.7	9.1
Proper harbour	2	4	9.1	18.2	100.0	9.1
Wharf	6	6	27.3	27.3	0.0	0.0
**Total atoll sample**	**22**					
**Dhaalu**						
Land reclamation	3	6	33.3	66.7	100.0	33.3
Proper harbour	1	2	11.1	22.2	100.0	11.1
Wharf	4	5	44.4	55.6	25.0	11.1
**Total atoll sample**	**9**					
**Thaa**						
Land reclamation	4	6	17.4	26.1	50.0	8.7
Proper harbour	4	5	17.4	21.7	25.0	4.3

Wharf	2	0	8.7	0.0	-100.0	-8.7
**Total atoll sample**	**23**					
**Laamu**						
Land reclamation	8	10	21.6	27.0	25.0	5.4
Proper harbour	7	9	18.9	24.3	28.6	5.4
Wharf	2	3	5.4	8.1	50.0	2.7
**Total atoll sample**	**37**					
**Gaafu Alifu-Dhaalu**						
Land reclamation	10	19	5.4	10.2	90.0	4.8
Proper harbour	10	16	5.4	8.6	60.0	3.2
Wharf	4	9	2.2	4.8	125.0	2.7
**Total atoll sample**	**186**					
**Gnaviyani**						
Land reclamation	1	1	100.0	100.0	0.0	0.0
Proper harbour	1	1	100.0	100.0	0.0	0.0
Wharf	0	0	0.0	0.0	0.0	0.0
**Total atoll sample**	**1**					
**Seenu**						
Land reclamation	7	9	25.9	33.3	28.6	7.4
Proper harbour	5	7	18.5	25.9	40.0	7.4
Wharf	3	3	11.1	11.1	0.0	0.0
**Total atoll sample**	**27**					

**TOTAL (all atolls)**	**Nb of islands**		**% (in proportion to the total atoll sample)**		**Rate of change in the number of equipped islands (+ X%), in proportion to the total island sample**	**Rate of change in the % of equipped islands (+ X%), in proportion to the total island sample**
	**2004-2006**	**2014-2016**	**2004-2006**	**2014-2016**		
Land reclamation	123	186	20.2	30.6	51.2	10.4
Proper harbour	96	149	15.8	24.5	55.2	8.7
Wharf	94	109	15.5	17.9	16.0	2.5
**Total island sample**	**608**					

Table B. Change in defence structures

	Nb of islands		% (in proportion to the number of islands per atoll)		Rate of change in the number of equipped islands (+ X%), in proportion to the number of islands per atoll	Rate of change in the % of equipped islands (+ X%), in proportion to the number of islands per atoll
	2004-2006	2014-2016	2004-2006	2014-2016		
**Ihavandhippolhu**						
**Type of defence structures**						
Longitudinal coastal	0	3	0.0	16.7	/	16.7
Transversal coastal	0	0	0.0	0.0	/	0.0
Long. and/or transv. marine	0	0	0.0	0.0	/	0.0
**Change in defence structures**						
Persistence of longitudinal coastal	0		0.0			
Additional longitudinal coastal	3		16.7			
Persistence of transversal coastal	0		0.0			
Additional transversal coastal	0		0.0			
Persistence of long. and/or transv. marine	0		0.0			
Additional long. and/or transv. marine	0		0.0			
**Total island sample (atoll scale)**	**18**					
**Haa Alifu-Noonu**						
**Type of defence structures**						
Longitudinal coastal	5	20	8.6	34.5	300.0	25.9
Transversal coastal	2	3	3.4	5.2	50.0	1.7
Long. and/or transv. marine	1	4	1.7	6.9	300.0	5.2
**Change in defence structures**						
Persistence of longitudinal coastal	4		6.9			
Additional longitudinal coastal	18		31.0			
Persistence of transversal coastal	1		1.7			
Additional transversal coastal	2		3.4			
Persistence of long. and/or transv. marine	1		1.7			
Additional long. and/or transv. marine	4		6.9			
**Total island sample (atoll scale)**	**58**					
**Maamakunudhoo**						
**Type of defence structures**						
Longitudinal coastal	1	1	50.0	50.0	0.0	0.0
Transversal coastal	0	0	0.0	0.0	/	0.0
Long. and/or transv. marine	0	0	0.0	0.0	/	0.0
**Change in defence structures**						

Persistence of longitudinal coastal	1		50.0			
Additional longitudinal coastal	1		50.0			
Persistence of transversal coastal	0		0.0			
Additional transversal coastal	0		0.0			
Persistence of long. and/or transv. marine	0		0.0			
Additional long. and/or transv. marine	0		0.0			
**Total island sample (atoll scale)**	**2**					
**Raa**						
**Type of defence structures**						
Longitudinal coastal	5	14	10.4	29.2	180.0	18.8
Transversal coastal	2	4	4.2	8.3	100.0	4.2
Long. and/or transv. marine	1	3	2.1	6.3	200.0	4.2
**Change in defence structures**						
Persistence of longitudinal coastal	4		8.3			
Additional longitudinal coastal	11		22.9			
Persistence of transversal coastal	2		4.2			
Additional transversal coastal	3		6.3			
Persistence of long. and/or transv. marine	1		2.1			
Additional long. and/or transv. marine	3		6.3			
**Total island sample (atoll scale)**	**48**					
**Lhaviyani**						
**Type of defence structures**						
Longitudinal coastal	3	6	11.5	23.1	100.0	11.5
Transversal coastal	1	7	3.8	26.9	600.0	23.1
Long. and/or transv. marine	2	3	7.7	11.5	50.0	3.8
**Change in defence structures**						
Persistence of longitudinal coastal	1		3.8			
Additional longitudinal coastal	6		23.1			
Persistence of transversal coastal	0		0.0			
Additional transversal coastal	7		26.9			
Persistence of long. and/or transv. marine	1		3.8			
Additional long. and/or transv. marine	3		11.5			
**Total island sample (atoll scale)**	**26**					
**Baa**						
**Type of defence structures**						
Longitudinal coastal	6	7	13.6	15.9	16,7	2.3
Transversal coastal	1	4	2.3	9.1	300,0	6.8
Long. and/or transv. marine	0	4	0.0	9.1	/	9.1
**Change in defence structures**						
Persistence of longitudinal coastal	3		6.8			
Additional longitudinal coastal	4		9.1			
Persistence of transversal coastal	0		0.0			

Additional transversal coastal	4		9.1			
Persistence of long. and/or transv. marine	0		0.0			
Additional long. and/or transv. marine	4		9.1			
**Total island sample (atoll scale)**	**44**					
**Kaashidhoo**						
**Type of defence structures**						
Longitudinal coastal	1	1	100.0	100.0	0,0	0.0
Transversal coastal	0	0	0.0	0.0	/	0.0
Long. and/or transv. marine	0	0	0.0	0.0	/	0.0
**Change in defence structures**						
Persistence of longitudinal coastal	1		100.0			
Additional longitudinal coastal	0		0.0			
Persistence of transversal coastal	0		0.0			
Additional transversal coastal	0		0.0			
Persistence of long. and/or transv. marine	0		0.0			
Additional long. and/or transv. marine	0		0.0			
**Total island sample (atoll scale)**	**1**					
**Goidhoo**						
**Type of defence structures**						
Longitudinal coastal	0	0	0.0	0.0	/	0.0
Transversal coastal	0	0	0.0	0.0	/	0.0
Long. and/or transv. marine	0	0	0.0	0.0	/	0.0
**Change in defence structures**						
Persistence of longitudinal coastal	0		0.0			
Additional longitudinal coastal	0		0.0			
Persistence of transversal coastal	0		0.0			
Additional transversal coastal	0		0.0			
Persistence of long. and/or transv. marine	0		0.0			
Additional long. and/or transv. marine	0		0.0			
**Total island sample (atoll scale)**	**1**					
**Gaafaru**						
**Type of defence structures**						
Longitudinal coastal	1	1	100.0	100.0	0,0	0.0
Transversal coastal	0	0	0.0	0.0	/	0.0
Long. and/or transv. marine	0	0	0.0	0.0	/	0.0
**Change in defence structures**						
Persistence of longitudinal coastal	1		100.0			
Additional longitudinal coastal	0		0.0			
Persistence of transversal coastal	0		0.0			
Additional transversal coastal	0		0.0			
Persistence of long. and/or transv. marine	0		0.0			
Additional long. and/or transv. marine	0		0.0			

**Total island sample (atoll scale)**	**1**					
**North Kaafu**						
**Type of defence structures**						
Longitudinal coastal	25	26	62.5	65.0	4.0	2.5
Transversal coastal	19	20	47.5	50.0	5.3	2.5
Long. and/or transv. marine	8	14	20.0	35.0	75.0	15.0
**Change in defence structures**						
Persistence of longitudinal coastal	20		50.0			
Additional longitudinal coastal	12		30.0			
Persistence of transversal coastal	15		37.5			
Additional transversal coastal	12		30.0			
Persistence of long. and/or transv. marine	8		20.0			
Additional long. and/or transv. marine	8		20.0			
**Total island sample (atoll scale)**	**40**					
**Thoddoo**						
**Type of defence structures**						
Longitudinal coastal	/	0	/	0.0	/	/
Transversal coastal	0	0	0.0	0.0	/	0.0
Long. and/or transv. marine	0	0	0.0	0.0	/	0.0
**Change in defence structures**						
Persistence of longitudinal coastal	/		/			
Additional longitudinal coastal	/		/			
Persistence of transversal coastal	0		0.0			
Additional transversal coastal	0		0.0			
Persistence of long. and/or transv. marine	0		0.0			
Additional long. and/or transv. marine	0		0.0			
**Total island sample (atoll scale)**	**1**					
**Rasdhoo**						
**Type of defence structures**						
Longitudinal coastal	2	2	33.3	33.3	0.0	0.0
Transversal coastal	3	4	50.0	66.7	33.3	16.7
Long. and/or transv. marine	3	2	50.0	33.3	-33.3	-16.7
**Change in defence structures**						
Persistence of longitudinal coastal	2		33.3			
Additional longitudinal coastal	2		33.3			
Persistence of transversal coastal	2		33.3			
Additional transversal coastal	2		33.3			
Persistence of long. and/or transv. marine	1		16.7			
Additional long. and/or transv. marine	2		33.3			
**Total island sample (atoll scale)**	**6**					
**Alifu**						
**Type of defence structures**						

Longitudinal coastal	3	6	16.7	33.3	100.0	16.7
Transversal coastal	8	9	44.4	50.0	12.5	5.6
Long. and/or transv. marine	5	7	27.8	38.9	40.0	11.1
**Change in defence structures**						
Persistence of longitudinal coastal	3		16.7			
Additional longitudinal coastal	6		33.3			
Persistence of transversal coastal	7		38.9			
Additional transversal coastal	6		33.3			
Persistence of long. and/or transv. marine	5		27.8			
Additional long. and/or transv. marine	3		16.7			
**Total island sample (atoll scale)**	**18**					
**South Kaafu**						
**Type of defence structures**						
Longitudinal coastal	12	16	50.0	66.7	33.3	16.7
Transversal coastal	12	15	50.0	62.5	25.0	12.5
Long. and/or transv. marine	0	5	0.0	20.8	/	20.8
**Change in defence structures**						
Persistence of longitudinal coastal	11		45.8			
Additional longitudinal coastal	10		41.7			
Persistence of transversal coastal	9		37.5			
Additional transversal coastal	9		37.5			
Persistence of long. and/or transv. marine	0		0.0			
Additional long. and/or transv. marine	5		20.8			
**Total island sample (atoll scale)**	**24**					
**Vaavu**						
**Type of defence structures**						
Longitudinal coastal	3	6	42.9	85.7	100.0	42.9
Transversal coastal	2	3	28.6	42.9	50.0	14.3
Long. and/or transv. marine	0	0	0.0	0.0	/	0.0
**Change in defence structures**						
Persistence of longitudinal coastal	2		28.6			
Additional longitudinal coastal	4		57.1			
Persistence of transversal coastal	2		28.6			
Additional transversal coastal	3		42.9			
Persistence of long. and/or transv. marine	0		0.0			
Additional long. and/or transv. marine	0		0.0			
**Total island sample (atoll scale)**	**7**					
**Faafu**						
**Type of defence structures**						
Longitudinal coastal	3	2	37.5	25.0	-33.3	-12.5
Transversal coastal	1	1	12.5	12.5	0.0	0.0
Long. and/or transv. marine	0	1	0.0	12.5	/	12.5

**Change in defence structures**						
Persistence of longitudinal coastal	2		25.0			
Additional longitudinal coastal	1		12.5			
Persistence of transversal coastal	1		12.5			
Additional transversal coastal	0		0.0			
Persistence of long. and/or transv. marine	0		0.0			
Additional long. and/or transv. marine	1		12.5			
**Total island sample (atoll scale)**	**8**					
**Meemu**						
**Type of defence structures**						
Longitudinal coastal	3	6	13.6	27.3	100.0	13.6
Transversal coastal	1	3	4.5	13.6	200.0	9.1
Long. and/or transv. marine	0	0	0.0	0.0	/	0.0
**Change in defence structures**						
Persistence of longitudinal coastal	3		13.6			
Additional longitudinal coastal	6		27.3			
Persistence of transversal coastal	1		4.5			
Additional transversal coastal	3		13.6			
Persistence of long. and/or transv. marine	0		0.0			
Additional long. and/or transv. marine	0		0.0			
**Total island sample (atoll scale)**	**22**					
**Dhaalu**						
**Type of defence structures**						
Longitudinal coastal	3	4	33.3	44.4	33.3	11.1
Transversal coastal	1	1	11.1	11.1	0.0	0.0
Long. and/or transv. marine	1	1	11.1	11.1	0.0	0.0
**Change in defence structures**						
Persistence of longitudinal coastal	3		33.3			
Additional longitudinal coastal	2		22.2			
Persistence of transversal coastal	1		11.1			
Additional transversal coastal	0		0.0			
Persistence of long. and/or transv. marine	1		11.1			
Additional long. and/or transv. marine	1		11.1			
**Total island sample (atoll scale)**	**9**					
**Thaa**						
**Type of defence structures**						
Longitudinal coastal	2	5	8.7	21.7	150.0	13.0
Transversal coastal	0	0	0.0	0.0	/	0.0
Long. and/or transv. marine	0	0	0.0	0.0	/	0.0
**Change in defence structures**						
Persistence of longitudinal coastal	2		8.7			
Additional longitudinal coastal	5		21.7			

Persistence of transversal coastal	0		0.0			
Additional transversal coastal	0		0.0			
Persistence of long. and/or transv. marine	0		0.0			
Additional long. and/or transv. marine	0		0.0			
**Total island sample (atoll scale)**	**23**					
**Laamu**						
**Type of defence structures**						
Longitudinal coastal	4	6	10.8	16.2	50.0	5.4
Transversal coastal	0	1	0.0	2.7	/	2.7
Long. and/or transv. marine	0	0	0.0	0.0	/	0.0
**Change in defence structures**						
Persistence of longitudinal coastal	1		2.7			
Additional longitudinal coastal	6		16.2			
Persistence of transversal coastal	0		0.0			
Additional transversal coastal	1		2.7			
Persistence of long. and/or transv. marine	0		0.0			
Additional long. and/or transv. marine	0		0.0			
**Total island sample (atoll scale)**	**37**					
**Gaafu Alifu-Dhaalu**						
**Type of defence structures**						
Longitudinal coastal	3	9	8.1	24.3	200.0	16.2
Transversal coastal	0	3	0.0	8.1	/	8.1
Long. and/or transv. marine	0	0	0.0	0.0	/	0.0
**Change in defence structures**						
Persistence of longitudinal coastal	0		0.0			
Additional longitudinal coastal	9		4.8			
Persistence of transversal coastal	0		0.0			
Additional transversal coastal	3		1.6			
Persistence of long. and/or transv. marine	0		0.0			
Additional long. and/or transv. marine	0		0.0			
**Total island sample (atoll scale)**	**186**					
**Gnaviyani**						
**Type of defence structures**						
Longitudinal coastal	0	1	0.0	100.0	/	100.0
Transversal coastal	0	0	0.0	0.0	/	0.0
Long. and/or transv. marine	0	1	0.0	100.0	/	100.0
**Change in defence structures**						
Persistence of longitudinal coastal	0		0.0			
Additional longitudinal coastal	1		100.0			
Persistence of transversal coastal	0		0.0			
Additional transversal coastal	0		0.0			
Persistence of long. and/or transv. marine	0		0.0			

Additional long. and/or transv. marine	1		100.0			
**Total island sample (atoll scale)**	**1**					
**Seenu**						
**Type of defence structures**						
Longitudinal coastal	4	6	14.8	22.2	50.0	7.4
Transversal coastal	1	2	3.7	7.4	100.0	3.7
Long. and/or transv. marine	0	0	0.0	0.0	/	0.0
**Change in defence structures**						
Persistence of longitudinal coastal	4		14.8			
Additional longitudinal coastal	5		18.5			
Persistence of transversal coastal	1		3.7			
Additional transversal coastal	1		3.7			
Persistence of long. and/or transv. marine	0		0.0			
Additional long. and/or transv. marine	0		0.0			
**Total island sample (atoll scale)**	**27**					

**TOTAL (all atolls)**						
**Type of defence structures**						
Longitudinal coastal	89	148	14.8	24.5	66.3	9.8
Transversal coastal	54	80	9.0	13.3	48.1	4.3
Long. and/or transv. marine	21	45	3.5	7.5	114.3	4.0
**Change in defence structures**						
Persistence of longitudinal coastal	68		11.3			
Additional longitudinal coastal	112		18.6			
Persistence of transversal coastal	42		7.0			
Additional transversal coastal	56		9.3			
Persistence of long. and/or transv. marine	18		3.0			
Additional long. and/or transv. marine	35		5.8			
**Total island sample (5 n.d. islands)**	**603**					

Table C. Change in shoreline type (per atoll)

Atoll			Entirely natural	Predominantely natural	Half-natural, half-fixed	Predominantely fixed	Entirely fixed	**Total island sample (atoll scale)**
**Ihavandhippolhu**								**18**
**Nb**	**2004-2006**		16	2	0	0	0	
	**2014-2016**		13	5	0	0	0	
**%**	**2004-2006**		88.9	11.1	0.0	0.0	0.0	
	**2014-2016**		72.2	27.8	0.0	0.0	0.0	
**Haa Alifu-Noonu**								**58**
**Nb**	**2004-2006**		49	9	0	0	0	
	**2014-2016**		33	23	1	1	0	
**%**	**2004-2006**		84.5	15.5	0.0	0.0	0.0	
	**2014-2016**		56.9	39.7	1.7	1.7	0.0	
**Maamakunudhoo**								**2**
**Nb**	**2004-2006**		1	1	0	0	0	
	**2014-2016**		1	1	0	0	0	
**%**	**2004-2006**		50.0	50.0	0.0	0.0	0.0	
	**2014-2016**		50.0	50.0	0.0	0.0	0.0	
**Raa**								**48**
**Nb**	**2004-2006**		40	7	0	0	1	
	**2014-2016**		34	12	0	1	1	
**%**	**2004-2006**		83.3	14.6	0.0	0.0	2.1	
	**2014-2016**		70.8	25.0	0.0	0.0	0.0	
**Lhaviyani**								**26**
**Nb**	**2004-2006**		21	3	0	0	2	
	**2014-2016**		16	8	1	0	1	
**%**	**2004-2006**		80.8	11.5	0.0	0.0	7.7	
	**2014-2016**		61.5	30.8	3.8	0.0	3.8	
**Baa**								**44**
**Nb**	**2004-2006**		35	7	0	2	0	
	**2014-2016**		33	7	0	4	0	
**%**	**2004-2006**		79.5	15.9	0.0	4.5	0.0	
	**2014-2016**		75.0	15.9	0.0	9.1	0.0	
**Kaashidhoo**								**1**
**Nb**	**2004-2006**		0	1	0	0	0	
	**2014-2016**		0	1	0	0	0	
**%**	**2004-2006**		0.0	100.0	0.0	0.0	0.0	
	**2014-2016**		0.0	100.0	0.0	0.0	0.0	
**Goidhoo**								**1**
**Nb**	**2004-2006**		1	0	0	0	0	
	**2014-2016**		1	0	0	0	0	
**%**	**2004-2006**		100.0	0.0	0.0	0.0	0.0	
	**2014-2016**		100.0	0.0	0.0	0.0	0.0	
**Gaafaru**								**1**
**Nb**	**2004-2006**		0	1	0	0	0	
	**2014-2016**		0	1	0	0	0	
**%**	**2004-2006**		0.0	100.0	0.0	0.0	0.0	
	**2014-2016**		0.0	100.0	0.0	0.0	0.0	
**North Kaafu**								**40**
**Nb**	**2004-2006**		9	14	3	9	5	

	2014-2016	6	13	3	13	5	
%	2004-2006	22.5	35.0	7.5	22.5	12.5	
	2014-2016	15.0	32.5	7.5	32.5	12.5	
**Thoddoo**							**1**
Nb	2004-2006	0	1	0	0	0	
	2014-2016	0	1	0	0	0	
%	2004-2006	0.0	100.0	0.0	0.0	0.0	
	2014-2016	0.0	100.0	0.0	0.0	0.0	
**Rasdhoo**							**6**
Nb	2004-2006	2	3	0	0	1	
	2014-2016	1	2	1	1	1	
%	2004-2006	33.3	35.0	7.5	22.5	12.5	
	2014-2016	16.7	32.5	7.5	32.5	12.5	
**Alifu**							**18**
Nb	2004-2006	10	7	1	0	0	
	2014-2016	9	6	3	0	0	
%	2004-2006	55.6	38.9	5.6	0.0	0.0	
	2014-2016	50.0	33.3	16.7	0.0	0.0	
**South Kaafu**							**24**
Nb	2004-2006	8	6	4	4	2	
	2014-2016	6	10	2	4	2	
%	2004-2006	33.3	25.0	16.7	16.7	8.3	
	2014-2016	25.0	41.7	8.3	16.7	8.3	
**Vaavu**							**7**
Nb	2004-2006	4	2	0	1	0	
	2014-2016	2	4	0	1	0	
%	2004-2006	57.1	28.6	0.0	14.3	0.0	
	2014-2016	28.6	57.1	0.0	14.3	0.0	
**Faafu**							**8**
Nb	2004-2006	6	2	0	0	0	
	2014-2016	5	1	1	1	0	
%	2004-2006	75.0	25.0	0.0	0.0	0.0	
	2014-2016	62.5	12.5	12.5	12.5	0.0	
**Meemu**							**22**
Nb	2004-2006	18	3	1	0	0	
	2014-2016	17	2	0	3	0	
%	2004-2006	81.8	13.6	4.5	0.0	0.0	
	2014-2016	77.3	9.1	0.0	13.6	0.0	
**Dhaalu**							**9**
Nb	2004-2006	6	1	0	0	2	
	2014-2016	2	4	0	1	2	
%	2004-2006	66.7	11.1	0.0	0.0	22.2	
	2014-2016	22.2	44.4	0.0	11.1	22.2	
**Thaa**							**23**
Nb	2004-2006	19	3	1	0	0	
	2014-2016	17	2	0	3	1	
%	2004-2006	82.6	13.0	4.3	0.0	0.0	
	2014-2016	73.9	8.7	0.0	13.0	4.3	
**Laamu**							**37**
Nb	2004-2006	29	8	0	0	0	
	2014-2016	27	9	1	0	0	
%	2004-2006	78.4	21.6	0.0	0.0	0.0	
	2014-2016	73.0	24.3	2.7	0.0	0.0	
**Gaafu Alifu-Dhaalu**							**186**
Nb	2004-2006	176	10	0	0	0	
	2014-2016	166	18	0	2	0	
%	2004-2006	94.6	5.4	0.0	0.0	0.0	
	2014-2016	89.2	9.7	0.0	1.1	0.0	

**Gnaviyani**							**1**
**Nb**	**2004-2006**	0	1	0	0	0	
	**2014-2016**	0	1	0	0	0	
**%**	**2004-2006**	0.0	100.0	0.0	0.0	0.0	
	**2014-2016**	0.0	100.0	0.0	0.0	0.0	
**Seenu**							**27**
**Nb**	**2004-2006**	22	3	2	0	0	
	**2014-2016**	18	6	1	0	2	
**%**	**2004-2006**	81.5	11.1	7.4	0.0	0.0	
	**2014-2016**	66.7	22.2	3.7	0.0	7.4	

**Total (all atolls)**							**608**
**Nb**	**2004-2006**	472	95	12	16	13	
	**2014-2016**	407	137	14	35	15	
**%**	**2004-2006**	77.6	15.6	2.0	2.6	2.1	
	**2014-2016**	66.9	22.5	2.3	5.8	2.5	

Table D. Change in shoreline type (all atolls)

Total (all atolls)							
		2014-2016					
		Number of islands					
		S(1)	S(2)	S(3)	S(4)	S(5)	Total
2004-2006	S(1)	406	58	3	4	1	472
	S(2)	1	77	3	13	1	95
	S(3)	0	0	7	4	1	12
	S(4)	0	1	0	14	1	16
	S(5)	0	1	1	0	11	13
	Total	407	137	14	35	15	608
		2014-2016					
		% of the atoll sample					
		S(1)	S(2)	S(3)	S(4)	S(5)	Total
2004-2006	S(1)	66.8	9.5	0.5	0.7	0.2	77.6
	S(2)	0.2	12.7	0.5	2.1	0.2	15.6
	S(3)	0.0	0.0	1.2	0.7	0.2	2.0
	S(4)	0.0	0.2	0.0	2.3	0.2	2.6
	S(5)	0.0	0.2	0.2	0.0	1.8	2.1
	Total	66.9	22.5	2.3	5.8	2.5	100.0

Nb	%	Change in human pressure
4	0.7	Decrease (from S2 to S1, from S3 to S1-2, from S4 to S1-3, etc.)
515	84.7	Stability (no change)
89	14.6	Increase (from S1 to S2-5, from S2 to S3-5, etc.)
608	100.0	Total

S3 – Change in inhabited island land area and population size, between 2004-2006 and 2014-2016

Content

Table A. Change in island land area
Table B. Change in population size
Table C. Correlation between change island land area and change in population size

Table A. Change in island land area

	Nb	%
(SA1). Land loss (≤–3%)	0	0.0
(SA2). Relative stability (±3%)	42	39.3
(SA3). Low increase (3≤x<10%)	30	28.0
(SA4). Moderate increase (10≤x<25%)	17	15.9
(SA5). High increase (25≤x<50%)	3	2.8
(SA6). Very high increase (50≤x<100%)	8	7.5
(SA7). Extremely high increase (x≥100%)	5	4.7
n.d.	2	1.9
TOTAL	107	100.0

Table B. Change in population size

	Nb	%
(POP1). Decrease (≤0%)	25	23.4
(POP2). Relative stability (<5%)	6	5.6
(POP3). Low increase (5≤x<10%)	5	4.7
(POP4). Moderate increase (10≤x<25%)	40	37.4
(POP5). high increase (25≤x<50%)	20	18.7
(POP6). Very high increase (50≤x<100%)	3	2.8
(POP7). Extremely high increase (x≥100%)	7	6.5
n.d.	1	0.9
TOTAL	107	100.0

Table C. Correlation between change island land area and change in population size

Total (all atolls)										
		2014-2016 (Number of islands)								
		POP1	POP2	POP3	POP4	POP5	POP6	POP7	n.d.	Total
2004-2006	SA1	0	0	0	0	0	0	0	-	0
	SA2	9	3	2	16	9	1	2	-	42
	SA3	9	1	3	13	3	0	1	-	30
	SA4	3	0	0	5	5	1	3	-	17
	SA5	0	0	0	1	2	0	0	-	3
	SA6	1	0	0	5	1	1	0	-	8
	SA7	3	1	0	0	0	0	1	-	5
	n.d.	-	-	-	-	-	-	-	2	2
	Total	25	5	5	40	20	3	7	2	107
		2014-2016 (% of the atoll sample)								
		POP1	POP2	POP3	POP4	POP5	POP6	POP7	n.d.	Total
2004-2006	SA1	0,0	0,0	0,0	0,0	0,0	0,0	0,0	-	0,0
	SA2	8,4	2,8	1,9	15,0	8,4	0,9	1,9	-	39,3
	SA3	8,4	0,9	2,8	12,1	2,8	0,0	0,9	-	28,0
	SA4	2,8	0,0	0,0	4,7	4,7	0,9	2,8	-	15,9
	SA5	0,0	0,0	0,0	0,9	1,9	0,0	0,0	-	2,8
	SA6	0,9	0,0	0,0	4,7	0,9	0,9	0,0	-	7,5
	SA7	2,8	0,9	0,0	0,0	0,0	0,0	0,9	-	4,7
	n.d.	-	-	-	-	-	-	-	1,9	1,9
	Total	23,4	4,7	4,7	37,4	18,7	2,8	6,5	1,9	100,0

Nb	%	Change
0	0,0	Land loss + decrease, stability or low increase in population
0	0,0	Land loss + increase (moderate to high) increase in population
0	0,0	Land loss + very high to extremely high increase in population
27	25,2	Relative stability or low increase in land area + decrease, stability or low increase in population
41	38,3	Relative stability or low increase in land area + increase (moderate to high) in population
4	3,7	Relative stability or low increase in land area + very high to extremely high increase in population
3	2,8	Increase (moderate to very high) in land area + decrease, stability or low increase in population
13	12,1	Increase (moderate to very high) in land area + increase (moderate to high) in population
4	3,7	Increase (moderate to very high) in land area + very high to extremely high increase in population
5	4,7	Extremely high increase in land area + decrease, stability or low increase in population
6	5,6	Extremely high increase in land area + increase (moderate to high) in population
2	1,9	Extremely high increase in land area + very high to extremely high increase in population
2	1,9	n.d.
**107**	**100.0**	**Total**

S4 – Change in human pressure exerted on the island's reef between 2004-2006 and 2014-2016

Content

Table A. Change in human pressure exerted on the island's reef
Table B. Change in the number of harbour basins per island (per atoll)

Table A. Change in human pressure exerted on the island's reef

	Nb of islands		% (in proportion to the number of islands per atoll)		Rate of change in the number of equipped islands (+ X%), in proportion to the number of islands per atoll	Rate of change in the % of equipped islands (+ X%), in proportion to the number of islands per atoll
	2004-2006	2014-2016	2004-2006	2014-2016		
**Ihavandhippolhu**						
**Type of human pressure on the island's reef**						
Boat channel(s)	3	7	16.7	38.9	133.3	22.2
Harbour basin(s)	2	4	11.1	22.2	100.0	11.1
Sediment dredging	0	1	0.0	5.6	/	5.6
**Origin of increased pressure**						
Extension of existing boat channel(s)	0		0.0			
Additional boat channel(s)	3		16.7			
Extension of existing harbour basin(s)	1		5.6			
Additional harbour basin(s)	2		11.1			
**Total island sample (atoll scale)**	**18**					
**Haa Alifu-Noonu**						
**Type of human pressure on the island's reef**						
Boat channel(s)	32	37	55.2	63.8	15.6	8.6
Harbour basin(s)	11	26	19.0	44.8	136.4	25.9
Sediment dredging	3	11	5.2	19.0	266.7	13.8
**Origin of increased pressure**						
Extension of existing boat channel(s)	1		1.7			
Additional boat channel(s)	20		34.5			
Extension of existing harbour basin(s)	4		6.9			
Additional harbour basin(s)	18		31.0			

**Total island sample (atoll scale)**	**58**					
**Maamakunudhoo**						
**Type of human pressure on the island's reef**						
Boat channel(s)	1	0	50.0	50.0	-100.0	0.0
Harbour basin(s)	1	1	50.0	50.0	0.0	0.0
Sediment dredging	1	2	50.0	50.0	100.0	0.0
**Origin of increased pressure**						
Extension of existing boat channel(s)	0		0.0			
Additional boat channel(s)	0		0.0			
Extension of existing harbour basin(s)	1		50.0			
Additional harbour basin(s)	1		50.0			
**Total island sample (atoll scale)**	**2**					
**Raa**						
**Type of human pressure on the island's reef**						
Boat channel(s)	16	16	33.3	33.3	0.0	0.0
Harbour basin(s)	6	14	12.5	29.2	133.3	16.7
Sediment dredging	3	5	6.3	10.4	66.7	4.2
**Origin of increased pressure**						
Extension of existing boat channel(s)	2		4.2			
Additional boat channel(s)	10		20.8			
Extension of existing harbour basin(s)	4		8.3			
Additional harbour basin(s)	11		22.9			
**Total island sample (atoll scale)**	**48**					
**Lhaviyani**						
**Type of human pressure on the island's reef**						
Boat channel(s)	6	7	23.1	26.9	16.7	3.8
Harbour basin(s)	5	6	19.2	23.1	20.0	3.8
Sediment dredging	6	7	23.1	26.9	16.7	3.8
**Origin of increased pressure**						
Extension of existing boat channel(s)	0		0.0			
Additional boat channel(s)	2		7.7			
Extension of existing harbour basin(s)	2		7.7			
Additional harbour basin(s)	2		7.7			
**Total island sample (atoll scale)**	**26**					
**Baa**						
**Type of human pressure on the island's reef**						
Boat channel(s)	23	22	52.3	50.0	-4.3	-2.3

Harbour basin(s)	7	16	15.9	36.4	128.6	20.5
Sediment dredging	7	9	15.9	20.5	28.6	4.5
**Origin of increased pressure**						
Extension of existing boat channel(s)	1		2.3			
Additional boat channel(s)	4		9.1			
Extension of existing harbour basin(s)	2		4.5			
Additional harbour basin(s)	7		15.9			
**Total island sample (atoll scale)**	**44**					
**Kaashidhoo**						
**Type of human pressure on the island's reef**						
Boat channel(s)	1	1	100.0	100.0	0.0	0.0
Harbour basin(s)	1	0	100.0	0.0	-100.0	-100.0
Sediment dredging	0	0	0.0	0.0	/	0.0
**Origin of increased pressure**						
Extension of existing boat channel(s)	0		0.0			
Additional boat channel(s)	0		0.0			
Extension of existing harbour basin(s)	0		0.0			
Additional harbour basin(s)	0		0.0			
**Total island sample (atoll scale)**	**1**					
**Goidhoo**						
**Type of human pressure on the island's reef**						
Boat channel(s)	0	0	0.0	0.0	/	0.0
Harbour basin(s)	1	1	100.0	100.0	0.0	0.0
Sediment dredging	0	0	0.0	0.0	/	0.0
**Origin of increased pressure**						
Extension of existing boat channel(s)	0		0.0			
Additional boat channel(s)	0		0.0			
Extension of existing harbour basin(s)	0		0.0			
Additional harbour basin(s)	0		0.0			
**Total island sample (atoll scale)**	**1**					
**Gaafaru**						
**Type of human pressure on the island's reef**						
Boat channel(s)	1	1	100.0	100.0	0.0	0.0
Harbour basin(s)	1	1	100.0	100.0	0.0	0.0
Sediment dredging	0	0	0.0	0.0	/	0.0
**Origin of increased pressure**						
Extension of existing boat channel(s)	0		0.0			

Additional boat channel(s)	0		0.0			
Extension of existing harbour basin(s)	1		100.0			
Additional harbour basin(s)	0		0.0			
**Total island sample (atoll scale)**	**1**					
**North Kaafu**						
**Type of human pressure on the island's reef**						
Boat channel(s)	26	25	65.0	62.5	-3.8	-2.5
Harbour basin(s)	21	24	52.5	60.0	14.3	7.5
Sediment dredging	17	22	42.5	55.0	29.4	12.5
**Origin of increased pressure**						
Extension of existing boat channel(s)	2		5.0			
Additional boat channel(s)	4		10.0			
Extension of existing harbour basin(s)	3		7.5			
Additional harbour basin(s)	2		5.0			
**Total island sample (atoll scale)**	**40**					
**Thoddoo**						
**Type of human pressure on the island's reef**						
Boat channel(s)	1	1	100.0	100.0	0.0	0.0
Harbour basin(s)	1	1	100.0	100.0	0.0	0.0
Sediment dredging	0	0	0.0	0.0	/	0.0
**Origin of increased pressure**						
Extension of existing boat channel(s)	0		0.0			
Additional boat channel(s)	2		200.0			
Extension of existing harbour basin(s)	0		0.0			
Additional harbour basin(s)	0		0.0			
**Total island sample (atoll scale)**	**1**					
**Rasdhoo**						
**Type of human pressure on the island's reef**						
Boat channel(s)	2	2	33.3	33.3	0.0	0.0
Harbour basin(s)	1	3	16.7	50.0	200.0	33.3
Sediment dredging	4	4	66.7	66.7	0.0	0.0
**Origin of increased pressure**						
Extension of existing boat channel(s)	0		0.0			
Additional boat channel(s)	1		16.7			
Extension of existing harbour basin(s)	0		0.0			
Additional harbour basin(s)	2		33.3			
**Total island sample (atoll scale)**	**6**					

**Alifu**						
**Type of human pressure on the island's reef**						
Boat channel(s)	16	16	88.9	88.9	0.0	0.0
Harbour basin(s)	8	9	44.4	50.0	12.5	5.6
Sediment dredging	4	7	22.2	38.9	75.0	16.7
**Origin of increased pressure**						
Extension of existing boat channel(s)	1		5.6			
Additional boat channel(s)	3		16.7			
Extension of existing harbour basin(s)	2		11.1			
Additional harbour basin(s)	1		5.6			
**Total island sample (atoll scale)**	**18**					
**South Kaafu**						
**Type of human pressure on the island's reef**						
Boat channel(s)	17	15	70.8	62.5	-11.8	-8.3
Harbour basin(s)	10	14	41.7	58.3	40.0	16.7
Sediment dredging	11	13	45.8	54.2	18.2	8.3
**Origin of increased pressure**						
Extension of existing boat channel(s)	1		4.2			
Additional boat channel(s)	5		20.8			
Extension of existing harbour basin(s)	2		8.3			
Additional harbour basin(s)	8		33.3			
**Total island sample (atoll scale)**	**24**					
**Vaavu**						
**Type of human pressure on the island's reef**						
Boat channel(s)	7	7	100.0	100.0	0.0	0.0
Harbour basin(s)	2	3	28.6	42.9	50.0	14.3
Sediment dredging	1	4	14.3	57.1	300.0	42.9
**Origin of increased pressure**						
Extension of existing boat channel(s)	0		0.0			
Additional boat channel(s)	1		14.3			
Extension of existing harbour basin(s)	0		0.0			
Additional harbour basin(s)	2		28.6			
**Total island sample (atoll scale)**	**7**					
**Faafu**						
**Type of human pressure on the island's reef**						
Boat channel(s)	5	4	62.5	50.0	-20.0	-12.5

Harbour basin(s)	1	3	12.5	37.5	200.0	25.0
Sediment dredging	1	1	12.5	12.5	0.0	0.0
**Origin of increased pressure**						
Extension of existing boat channel(s)	0		0.0			
Additional boat channel(s)	1		12.5			
Extension of existing harbour basin(s)	0		0.0			
Additional harbour basin(s)	2		25.0			
**Total island sample (atoll scale)**	**8**					
**Meemu**						
**Type of human pressure on the island's reef**						
Boat channel(s)	5	5	22.7	22.7	0.0	0.0
Harbour basin(s)	3	5	13.6	22.7	66.7	9.1
Sediment dredging	3	5	13.6	22.7	66.7	9.1
**Origin of increased pressure**						
Extension of existing boat channel(s)	1		4.5			
Additional boat channel(s)	1		4.5			
Extension of existing harbour basin(s)	1		4.5			
Additional harbour basin(s)	3		13.6			
**Total island sample (atoll scale)**	**22**					
**Dhaalu**						
**Type of human pressure on the island's reef**						
Boat channel(s)	7	6	77.8	66.7	-14.3	-11.1
Harbour basin(s)	1	4	11.1	44.4	300.0	33.3
Sediment dredging	4	4	44.4	44.4	0.0	0.0
**Origin of increased pressure**						
Extension of existing boat channel(s)	1		11.1			
Additional boat channel(s)	1		11.1			
Extension of existing harbour basin(s)	0		0.0			
Additional harbour basin(s)	3		33.3			
**Total island sample (atoll scale)**	**9**					
**Thaa**						
**Type of human pressure on the island's reef**						
Boat channel(s)	6	5	26.1	21.7	-16.7	-4.3
Harbour basin(s)	4	5	17.4	21.7	25.0	4.3
Sediment dredging	2	3	8.7	13.0	50.0	4.3
**Origin of increased pressure**						
Extension of existing boat channel(s)	2		8.7			

Additional boat channel(s)	1		4.3			
Extension of existing harbour basin(s)	19		82.6			
Additional harbour basin(s)	1		4.3			
**Total island sample (atoll scale)**	**23**					
**Laamu**						
**Type of human pressure on the island's reef**						
Boat channel(s)	7	8	18.9	21.6	14.3	2.7
Harbour basin(s)	8	10	21.6	27.0	25.0	5.4
Sediment dredging	4	6	10.8	16.2	50.0	5.4
**Origin of increased pressure**						
Extension of existing boat channel(s)	0		0.0			
Additional boat channel(s)	3		8.1			
Extension of existing harbour basin(s)	5		13.5			
Additional harbour basin(s)	3		8.1			
**Total island sample (atoll scale)**	**37**					
**Gaafu Alifu-Dhaalu**						
**Type of human pressure on the island's reef**						
Boat channel(s)	16	24	8.6	12.9	50.0	4.3
Harbour basin(s)	9	20	4.8	10.8	122.2	5.9
Sediment dredging	7	14	3.8	7.5	100.0	3.8
**Origin of increased pressure**						
Extension of existing boat channel(s)	1		0.5			
Additional boat channel(s)	11		5.9			
Extension of existing harbour basin(s)	6		3.2			
Additional harbour basin(s)	12		6.5			
**Total island sample (atoll scale)**	**186**					
**Gnaviyani**						
**Type of human pressure on the island's reef**						
Boat channel(s)	1	1	100.0	100.0	0.0	0.0
Harbour basin(s)	1	1	100.0	100.0	0.0	0.0
Sediment dredging	0	0	0.0	0.0	/	0.0
**Origin of increased pressure**						
Extension of existing boat channel(s)	0		0.0			
Additional boat channel(s)	0		0.0			
Extension of existing harbour basin(s)	0		0.0			
Additional harbour basin(s)	0		0.0			
**Total island sample (atoll scale)**	**1**					

**Seenu**						
**Type of human pressure on the island's reef**						
Boat channel(s)	10	13	37.0	48.1	30.0	11.1
Harbour basin(s)	6	7	22.2	25.9	16.7	3.7
Sediment dredging	6	7	22.2	25.9	16.7	3.7
**Origin of increased pressure**						
Extension of existing boat channel(s)	1		3.7			
Additional boat channel(s)	5		18.5			
Extension of existing harbour basin(s)	2		7.4			
Additional harbour basin(s)	4		14.8			
**Total island sample (atoll scale)**	**27**					

**TOTAL (all atolls)**						
**Type of human pressure on the island's reef**						
Boat channel(s)	209	223	34.7	37.0	6.7	2.3
Harbour basin(s)	110	178	18.2	29.5	61.8	11.3
Sediment dredging	84	125	13.9	20.7	48.8	6.8
**Origin of increased pressure**						
Extension of existing boat channel(s)	14		2.3			
Additional boat channel(s)	77		12.8			
Extension of existing harbour basin(s)	38		6.3			
Additional harbour basin(s)	84		13.9			
**Total island sample (5 n.d. islands)**	**603**					

Table B. Change in the number of harbour basins per island (per atoll)

Ihavandhippolhu

		2014-2016 Number of islands							2014-2016 % of the atoll sample				
		**0**	**1**	**2**	**≥3**	**Total**			**0**	**1**	**2**	**≥3**	**Total**
**2004-2006**	**0**	14	2	0	0	16	**2004-2006**	**0**	77.8	11.1	0.0	0.0	88.9
	**1**	0	2	0	0	2		**1**	0.0	11.1	0.0	0.0	11.1
	**2**	0	0	0	0	0		**2**	0.0	0.0	0.0	0.0	0.0
	**≥3**	0	0	0	0	0		**≥3**	0.0	0.0	0.0	0.0	0.0
	**Total**	14	4	0	0	18		**Total**	77.8	22.2	0.0	0.0	100.0

Nb	%	Change in human pressure
0	0.0	Decrease (from S2 to S1, from S3 to S1-2, from S4 to S1-3, etc.)
16	88.9	Stability (no change)
2	11.1	Increase (from S1 to S2-5, from S2 to S3-5, etc.)
18	100.0	Total

Haa Alifu-Noonu

		2014-2016 Number of islands							2014-2016 % of the atoll sample				
		**0**	**1**	**2**	**≥3**	**Total**			**0**	**1**	**2**	**≥3**	**Total**
**2004-2006**	**0**	32	11	4	0	47	**2004-2006**	**0**	55.2	19.0	6.9	0.0	81.0
	**1**	0	6	3	0	9		**1**	0.0	10.3	5.2	0.0	15.5
	**2**	0	0	2	0	2		**2**	0.0	0.0	3.4	0.0	3.4
	**≥3**	0	0	0	0	0		**≥3**	0.0	0.0	0.0	0.0	0.0
	**Total**	32	17	9	0	58		**Total**	55.2	29.3	15.5	0.0	100.0

Nb	%	Change in human pressure
0	0.0	Decrease (from S2 to S1, from S3 to S1-2, from S4 to S1-3, etc.)
40	69.0	Stability (no change)
18	31.0	Increase (from S1 to S2-5, from S2 to S3-5, etc.)
58	100.0	Total

Maamakunudhoo

		2014-2016 Number of islands				
		0	1	2	≥3	Total
2004-2006	0	1	0	0	0	1
	1	0	0	1	0	1
	2	0	0	0	0	0
	≥3	0	0	0	0	0
	Total	1	0	1	0	2

		2014-2016 % of the atoll sample				
		0	1	2	≥3	Total
2004-2006	0	50.0	0.0	0.0	0.0	50.0
	1	0.0	0.0	50.0	0.0	50.0
	2	0.0	0.0	0.0	0.0	0.0
	≥3	0.0	0.0	0.0	0.0	0.0
	Total	50.0	0.0	50.0	0.0	100.0

Nb	%	Change in human pressure
0	0.0	Decrease (from S2 to S1, from S3 to S1-2, from S4 to S1-3, etc.)
1	50.0	Stability (no change)
1	50.0	Increase (from S1 to S2-5, from S2 to S3-5, etc.)
2	100.0	Total

Raa

		2014-2016 Number of islands				
		0	1	2	≥3	Total
2004-2006	0	34	6	2	0	42
	1	0	3	2	1	6
	2	0	0	0	0	0
	≥3	0	0	0	0	0
	Total	34	9	4	1	48

		2014-2016 % of the atoll sample				
		0	1	2	≥3	Total
2004-2006	0	70.8	12.5	4.2	0.0	87.5
	1	0.0	6.3	4.2	2.1	12.5
	2	0.0	0.0	0.0	0.0	0.0
	≥3	0.0	0.0	0.0	0.0	0.0
	Total	70.8	18.8	8.3	2.1	100.0

Nb	%	Change in human pressure
0	0.0	Decrease (from S2 to S1, from S3 to S1-2, from S4 to S1-3, etc.)
37	77.1	Stability (no change)
11	22.9	Increase (from S1 to S2-5, from S2 to S3-5, etc.)
48	100.0	Total

Lhaviyani

		2014-2016 Number of islands							2014-2016 % of the atoll sample				
		0	1	2	≥3	Total			0	1	2	≥3	Total
2004-2006	0	20	1	0	0	21	2004-2006	0	76.9	3.8	0.0	0.0	80.8
	1	0	3	1	0	4		1	0.0	11.5	3.8	0.0	15.4
	2	0	0	1	0	1		2	0.0	0.0	3.8	0.0	3.8
	≥3	0	0	0	0	0		≥3	0.0	0.0	0.0	0.0	0.0
	Total	20	4	2	0	26		Total	76.9	15.4	7.7	0.0	100.0

Nb	%	Change in human pressure
0	0.0	Decrease (from S2 to S1, from S3 to S1-2, from S4 to S1-3, etc.)
24	92.3	Stability (no change)
2	7.7	Increase (from S1 to S2-5, from S2 to S3-5, etc.)
26	100.0	Total

Baa

		2014-2016 Number of islands							2014-2016 % of the atoll sample				
		0	1	2	≥3	Total			0	1	2	≥3	Total
2004-2006	0	28	8	1	0	37	2004-2006	0	63.6	18.2	2.3	0.0	84.1
	1	0	4	0	0	4		1	0.0	9.1	0.0	0.0	9.1
	2	0	1	2	0	3		2	0.0	2.3	4.5	0.0	6.8
	≥3	0	0	0	0	0		≥3	0.0	0.0	0.0	0.0	0.0
	Total	28	13	3	0	44		Total	63.6	29.5	6.8	0.0	100.0

Nb	%	Change in human pressure
1	2.3	Decrease (from S2 to S1, from S3 to S1-2, from S4 to S1-3, etc.)
34	77.3	Stability (no change)
9	20.5	Increase (from S1 to S2-5, from S2 to S3-5, etc.)
44	100.0	Total

Kaashidhoo

		2014-2016 Number of islands				
		0	1	2	≥3	Total
2004-2006	0	0	0	0	0	0
	1	0	1	0	0	1
	2	0	0	0	0	0
	≥3	0	0	0	0	0
	Total	0	1	0	0	1

		2014-2016 % of the atoll sample				
		0	1	2	≥3	Total
2004-2006	0	0.0	0.0	0.0	0.0	0.0
	1	0.0	100.0	0.0	0.0	100.0
	2	0.0	0.0	0.0	0.0	0.0
	≥3	0.0	0.0	0.0	0.0	0.0
	Total	0.0	100.0	0.0	0.0	100.0

Nb	%	Change in human pressure
0	0.0	Decrease (from S2 to S1, from S3 to S1-2, from S4 to S1-3, etc.)
1	100.0	Stability (no change)
0	0.0	Increase (from S1 to S2-5, from S2 to S3-5, etc.)
1	100.0	Total

Goidhoo

		2014-2016 Number of islands				
		0	1	2	≥3	Total
2004-2006	0	1	0	0	0	1
	1	0	0	0	0	0
	2	0	0	0	0	0
	≥3	0	0	0	0	0
	Total	1	0	0	0	1

		2014-2016 % of the atoll sample				
		0	1	2	≥3	Total
2004-2006	0	100.0	0.0	0.0	0.0	100.0
	1	0.0	0.0	0.0	0.0	0.0
	2	0.0	0.0	0.0	0.0	0.0
	≥3	0.0	0.0	0.0	0.0	0.0
	Total	100.0	0.0	0.0	0.0	100.0

Nb	%	Change in human pressure
0	0.0	Decrease (from S2 to S1, from S3 to S1-2, from S4 to S1-3, etc.)
1	100.0	Stability (no change)
0	0.0	Increase (from S1 to S2-5, from S2 to S3-5, etc.)
1	100.0	Total

Gaafaru

		2014-2016 Number of islands							2014-2016 % of the atoll sample				
		0	1	2	≥3	Total			0	1	2	≥3	Total
2004-2006	0	0	0	0	0	0	2004-2006	0	0.0	0.0	0.0	0.0	0.0
	1	0	1	0	0	1		1	0.0	100.0	0.0	0.0	100.0
	2	0	0	0	0	0		2	0.0	0.0	0.0	0.0	0.0
	≥3	0	0	0	0	0		≥3	0.0	0.0	0.0	0.0	0.0
	Total	0	1	0	0	1		Total	0.0	100.0	0.0	0.0	100.0

Nb	%	Change in human pressure
0	0.0	Decrease (from S2 to S1, from S3 to S1-2, from S4 to S1-3, etc.)
1	100.0	Stability (no change)
0	0.0	Increase (from S1 to S2-5, from S2 to S3-5, etc.)
1	100.0	Total

North Kaafu

		2014-2016 Number of islands							2014-2016 % of the atoll sample				
		0	1	2	≥3	Total			0	1	2	≥3	Total
2004-2006	0	16	3	0	0	19	2004-2006	0	40.0	7.5	0.0	0.0	47.5
	1	0	9	1	0	10		1	0.0	22.5	2.5	0.0	25.0
	2	0	0	6	1	7		2	0.0	0.0	15.0	2.5	17.5
	≥3	0	0	0	4	4		≥3	0.0	0.0	0.0	10.0	10.0
	Total	16	12	7	5	40		Total	40.0	30.0	17.5	12.5	100.0

Nb	%	Change in human pressure
0	0.0	Decrease (from S2 to S1, from S3 to S1-2, from S4 to S1-3, etc.)
35	87.5	Stability (no change)
5	12.5	Increase (from S1 to S2-5, from S2 to S3-5, etc.)
40	100.0	Total

Thoddoo														
		2014-2016							2014-2016				
		Number of islands							% of the atoll sample				
		0	1	2	≥3	Total			0	1	2	≥3	Total
2004-2006	0	0	0	0	0	0	2004-2006	0	0.0	0.0	0.0	0.0	0.0
	1	0	1	0	0	1		1	0.0	100.0	0.0	0.0	100.0
	2	0	0	0	0	0		2	0.0	0.0	0.0	0.0	0.0
	≥3	0	0	0	0	0		≥3	0.0	0.0	0.0	0.0	0.0
	Total	0	1	0	0	1		Total	0.0	100.0	0.0	0.0	100.0

Nb	%	Change in human pressure
0	0.0	Decrease (from S2 to S1, from S3 to S1-2, from S4 to S1-3, etc.)
1	100.0	Stability (no change)
0	0.0	Increase (from S1 to S2-5, from S2 to S3-5, etc.)
1	100.0	Total

Rasdhoo														
		2014-2016							2014-2016				
		Number of islands							% of the atoll sample				
		0	1	2	≥3	Total			0	1	2	≥3	Total
2004-2006	0	3	2	0	0	5	2004-2006	0	50.0	33.3	0.0	0.0	83.3
	1	0	1	0	0	1		1	0.0	16.7	0.0	0.0	16.7
	2	0	0	0	0	0		2	0.0	0.0	0.0	0.0	0.0
	≥3	0	0	0	0	0		≥3	0.0	0.0	0.0	0.0	0.0
	Total	3	3	0	0	6		Total	50.0	50.0	0.0	0.0	100.0

Nb	%	Change in human pressure
0	0.0	Decrease (from S2 to S1, from S3 to S1-2, from S4 to S1-3, etc.)
4	66.7	Stability (no change)
2	33.3	Increase (from S1 to S2-5, from S2 to S3-5, etc.)
6	100.0	Total

Alifu

		2014-2016 Number of islands							2014-2016 % of the atoll sample				
		**0**	**1**	**2**	**≥3**	**Total**			**0**	**1**	**2**	**≥3**	**Total**
**2004-2006**	**0**	9	1	0	0	10	**2004-2006**	**0**	50.0	5.6	0.0	0.0	55.6
	**1**	0	5	0	0	5		**1**	0.0	27.8	0.0	0.0	27.8
	**2**	0	0	2	0	2		**2**	0.0	0.0	11.1	0.0	11.1
	**≥3**	0	0	0	1	1		**≥3**	0.0	0.0	0.0	5.6	5.6
	**Total**	9	6	2	1	18		**Total**	50.0	33.3	11.1	5.6	100.0

Nb	%	Change in human pressure
0	0.0	Decrease (from S2 to S1, from S3 to S1-2, from S4 to S1-3, etc.)
17	94.4	Stability (no change)
1	5.6	Increase (from S1 to S2-5, from S2 to S3-5, etc.)
18	100.0	Total

South Kaafu

		2014-2016 Number of islands							2014-2016 % of the atoll sample				
		**0**	**1**	**2**	**≥3**	**Total**			**0**	**1**	**2**	**≥3**	**Total**
**2004-2006**	**0**	10	3	1	0	14	**2004-2006**	**0**	41.7	12.5	4.2	0.0	58.3
	**1**	0	4	0	2	6		**1**	0.0	16.7	0.0	8.3	25.0
	**2**	0	0	1	1	2		**2**	0.0	0.0	4.2	4.2	8.3
	**≥3**	0	0	1	1	2		**≥3**	0.0	0.0	4.2	4.2	8.3
	**Total**	10	7	3	4	24		**Total**	41.7	29.2	12.5	16.7	100.0

Nb	%	Change in human pressure
1	4.2	Decrease (from S2 to S1, from S3 to S1-2, from S4 to S1-3, etc.)
16	66.7	Stability (no change)
7	29.2	Increase (from S1 to S2-5, from S2 to S3-5, etc.)
24	100.0	Total

Vaavu

		2014-2016 Number of islands				
		0	1	2	≥3	Total
2004-2006	0	4	1	0	0	5
	1	0	0	1	0	1
	2	0	0	1	0	1
	≥3	0	0	0	0	0
	Total	4	1	2	0	7

		2014-2016 % of the atoll sample				
		0	1	2	≥3	Total
2004-2006	0	57.1	14.3	0.0	0.0	71.4
	1	0.0	0.0	14.3	0.0	14.3
	2	0.0	0.0	14.3	0.0	14.3
	≥3	0.0	0.0	0.0	0.0	0.0
	Total	57.1	14.3	28.6	0.0	100.0

Nb	%	Change in human pressure
0	0.0	Decrease (from S2 to S1, from S3 to S1-2, from S4 to S1-3, etc.)
5	71.4	Stability (no change)
2	28.6	Increase (from S1 to S2-5, from S2 to S3-5, etc.)
7	100.0	Total

Faafu

		2014-2016 Number of islands				
		0	1	2	≥3	Total
2004-2006	0	5	1	1	0	7
	1	0	0	0	0	0
	2	0	0	1	0	1
	≥3	0	0	0	0	0
	Total	5	1	2	0	8

		2014-2016 % of the atoll sample				
		0	1	2	≥3	Total
2004-2006	0	62.5	12.5	12.5	0.0	87.5
	1	0.0	0.0	0.0	0.0	0.0
	2	0.0	0.0	1.5	0.0	12.5
	≥3	0.0	0.0	0.0	0.0	0.0
	Total	62.5	12.5	25.0	0.0	100.0

Nb	%	Change in human pressure
0	0.0	Decrease (from S2 to S1, from S3 to S1-2, from S4 to S1-3, etc.)
6	75.0	Stability (no change)
2	25.0	Increase (from S1 to S2-5, from S2 to S3-5, etc.)
8	100.0	Total

Meemu

		2014-2016 Number of islands							2014-2016 % of the atoll sample				
		0	1	2	≥3	Total			0	1	2	≥3	Total
2004-2006	0	17	1	1	0	19	2004-2006	0	77.3	4.5	4.5	0.0	86.4
	1	0	1	0	0	1		1	0.0	4.5	0.0	0.0	4.5
	2	0	0	0	1	1		2	0.0	0.0	0.0	4.5	4.5
	≥3	0	0	0	1	1		≥3	0.0	0.0	0.0	4.5	4.5
	Total	17	2	1	2	22		Total	77.3	9.1	4.5	9.1	100.0

Nb	%	Change in human pressure
0	0.0	Decrease (from S2 to S1, from S3 to S1-2, from S4 to S1-3, etc.)
19	86.4	Stability (no change)
3	13.6	Increase (from S1 to S2-5, from S2 to S3-5, etc.)
22	100.0	Total

Dhaalu

		2014-2016 Number of islands							2014-2016 % of the atoll sample				
		0	1	2	≥3	Total			0	1	2	≥3	Total
2004-2006	0	5	2	1	0	8	2004-2006	0	55.6	22.2	11.1	0.0	88.9
	1	0	1	0	0	1		1	0.0	11.1	0.0	0.0	11.1
	2	0	0	0	0	0		2	0.0	0.0	0.0	0.0	0.0
	≥3	0	0	0	0	0		≥3	0.0	0.0	0.0	0.0	0.0
	Total	5	3	1	0	9		Total	55.6	33.3	11.1	0.0	100.0

Nb	%	Change in human pressure
0	0.0	Decrease (from S2 to S1, from S3 to S1-2, from S4 to S1-3, etc.)
6	66.7	Stability (no change)
3	33.3	Increase (from S1 to S2-5, from S2 to S3-5, etc.)
9	100.0	Total

Thaa

		2014-2016 Number of islands							2014-2016 % of the atoll sample				
		0	1	2	≥3	Total			0	1	2	≥3	Total
2004-2006	0	18	1	0	0	19	2004-2006	0	78.3	4.3	0.0	0.0	82.6
	1	0	1	0	0	1		1	0.0	4.3	0.0	0.0	4.3
	2	0	1	1	0	2		2	0.0	4.3	4.3	0.0	8.7
	≥3	0	0	0	1	1		≥3	0.0	0.0	0.0	4.3	4.3
	Total	18	3	1	1	23		Total	78.3	13.0	4.3	4.3	100.0

Nb	%	Change in human pressure
1	4.3	Decrease (from S2 to S1, from S3 to S1-2, from S4 to S1-3, etc.)
21	91.3	Stability (no change)
1	4.3	Increase (from S1 to S2-5, from S2 to S3-5, etc.)
23	100.0	Total

Laamu

		2014-2016 Number of islands							2014-2016 % of the atoll sample				
		0	1	2	≥3	Total			0	1	2	≥3	Total
2004-2006	0	27	2	0	0	29	2004-2006	0	73.0	5.4	0.0	0.0	78.4
	1	0	5	0	1	6		1	0.0	13.5	0.0	2.7	16.2
	2	0	1	0	0	1		2	0.0	2.7	0.0	0.0	2.7
	≥3	0	0	0	1	1		≥3	0.0	0.0	0.0	2.7	2.7
	Total	27	8	0	2	37		Total	73.0	21.6	0.0	5.4	100.0

Nb	%	Change in human pressure
1	2.7	Decrease (from S2 to S1, from S3 to S1-2, from S4 to S1-3, etc.)
33	89.2	Stability (no change)
3	8.1	Increase (from S1 to S2-5, from S2 to S3-5, etc.)
37	100.0	Total

Gaafu Alifu-Dhaalu															
		**2014-2016**							**2014-2016**				
		**Number of islands**							**% of the atoll sample**				
		**0**	**1**	**2**	**≥3**	**Total**			**0**	**1**	**2**	**≥3**	**Total**
**2004-2006**	**0**	166	10	1	0	177	**2004-2006**	**0**	89.2	5.4	0.5	0.0	95.2
	**1**	0	6	1	0	7		**1**	0.0	3.2	0.5	0.0	3.8
	**2**	0	0	1	0	1		**2**	0.0	0.0	0.5	0.0	0.5
	**≥3**	0	0	0	1	1		**≥3**	0.0	0.0	0.0	0.5	0.5
	**Total**	166	16	3	1	186		**Total**	89.2	8.6	1.6	0.5	100.0

Nb	%	Change in human pressure
0	0.0	Decrease (from S2 to S1, from S3 to S1-2, from S4 to S1-3, etc.)
174	93.5	Stability (no change)
12	6.5	Increase (from S1 to S2-5, from S2 to S3-5, etc.)
186	100.0	Total

Gnaviyani													
		**2014-2016**							**2014-2016**				
		**Number of islands**							**% of the atoll sample**				
		**0**	**1**	**2**	**≥3**	**Total**			**0**	**1**	**2**	**≥3**	**Total**
**2004-2006**	**0**	1	0	0	0	1	**2004-2006**	**0**	100.0	0.0	0.0	0.0	0.5
	**1**	0	0	0	0	0		**1**	0.0	0.0	0.0	0.0	0.0
	**2**	0	0	0	0	0		**2**	0.0	0.0	0.0	0.0	0.0
	**≥3**	0	0	0	0	0		**≥3**	0.0	0.0	0.0	0.0	0.0
	**Total**	1	0	0	0	1		**Total**	100.0	0.0	0.0	0.0	100.0

Nb	%	Change in human pressure
0	0.0	Decrease (from S2 to S1, from S3 to S1-2, from S4 to S1-3, etc.)
1	100.0	Stability (no change)
0	0.0	Increase (from S1 to S2-5, from S2 to S3-5, etc.)
1	100.0	Total

Seenu

		2014-2016 Number of islands							2014-2016 % of the atoll sample				
		0	1	2	≥3	Total			0	1	2	≥3	Total
2004-2006	0	21	0	0	0	21	2004-2006	0	77.8	0.0	0.0	0.0	11.3
	1	0	4	0	0	4		1	0.0	14.8	0.0	0.0	2.2
	2	0	0	1	0	1		2	0.0	0.0	3.7	0.0	0.5
	≥3	0	0	0	1	1		≥3	0.0	0.0	0.0	3.7	0.5
	Total	21	4	1	1	27		Total	77.8	14.8	3.7	3.7	100.0

Nb	%	Change in human pressure
0	0.0	Decrease (from S2 to S1, from S3 to S1-2, from S4 to S1-3, etc.)
27	100.0	Stability (no change)
0	0.0	Increase (from S1 to S2-5, from S2 to S3-5, etc.)
27	100.0	Total

Total (all atolls)

		2014-2016 Number of islands							2014-2016 % of the atoll sample				
		0	1	2	≥3	Total			0	1	2	≥3	Total
2004-2006	0	430	55	12	1	498	2004-2006	0	70.7	9.0	2.0	0.2	81.9
	1	0	55	11	4	70		1	0.0	9.0	1.8	0.7	11.5
	2	0	4	19	5	28		2	0.0	0.7	3.1	0.8	4.6
	≥3	0	0	1	11	12		≥3	0.0	0.0	0.2	1.8	2.0
	Total	430	114	43	21	608		Total	70.7	18.8	7.1	3.5	100.0

Nb	%	Change in human pressure
5	0.8	Decrease (from S2 to S1, from S3 to S1-2, from S4 to S1-3, etc.)
515	84.7	Stability (no change)
88	14.5	Increase (from S1 to S2-5, from S2 to S3-5, etc.)
608	100.0	Total

S5 – Change in island type between 2004-2006 and 2014-2016

Content

Table A. Change in shoreline type (S)
Table B. Change in human pressure exerted on the island's reef (R)
Table C. Island type (T), based on the combination of shoreline type (S) with human pressure exerted on the island's reef (R)
Table D. Change in island type between 2004-2006 and 2014-2016

Table A. Change in shoreline type (S)

(S1). Entirely natural = No modification
(S2). Predominantly natural = Limited modification
(S3). Half-natural, half-fixed = Substantial modification
(S4). Predominantly fixed = Very substantial modification
(S5). Entirely fixed = Complete modification

Total (all atolls)							
		2014-2016					
		Number of islands					
		S1	S2	S3	S4	S5	Total
2004-2006	S1	406	58	3	4	1	472
	S2	1	77	3	13	1	95
	S3	0	0	7	4	1	12
	S4	0	1	0	14	1	16
	S5	0	1	1	0	11	13
	Total	407	137	14	35	15	608
		2014-2016					
		% of the atoll sample					
		S1	S2	S3	S4	S5	Total
2004-2006	S1	66,8	9.5	0.5	0.7	0.2	77.6
	S2	0.2	12.7	0.5	2.1	0.2	15.6
	S3	0.0	0.0	1.2	0.7	0.2	2.0
	S4	0.0	0.2	0.0	2.3	0.2	2.6
	S5	0.0	0.2	0.2	0.0	1.8	2.1
	Total	66.9	22.5	2.3	5.8	2.5	100.0

Nb	%	Change in human pressure
4	0.7	Decrease
515	84.7	Stability (no change)
89	14.6	Increase
608	100.0	Total

Table B. Change in human pressure exerted on the island's reef (R)

**Human pressure exerted on the island's reef (R)**	**Categories (2004-2006)**	**Categories (2014-2016)**
(R1) – No to limited pressure	• 0 harbour basin + 0 channel + 0 dredging + 0 marine structure	
(R2) – Moderate pressure	• 0 harbour basin + ≥1 channel<br>• 0 harbour basin + ≥1 dredging<br>• 0 harbour basin + ≥1 marine structure<br>• 1 harbour basin + 0 Channel/Dredging/Marine structure<br>• 1 harbour basin + ≥1 channel<br>• 1 harbour basin + ≥1 dredging<br>• 1 harbour basin + ≥1 marine structure	• 0 harbour basin + persistence of ≥1 channel<br>• 0 harbour basin + extension of ≥1 channel<br>• 0 harbour basin + ≥1 new/additional channel<br>• 0 harbour basin + ≥1 dredging<br>• 0 harbour basin + persistence of ≥1 marine structure<br>• 0 harbour basin + ≥1 new/additional marine structure<br>• Persistence of 1 harbour basin (+ 0 or >=1 channel/Dredge/Marine structure)<br>• Extension of 1 harbour basin (+ 0 or >=1 channel/Dredge/Marine structure)<br>• 1 new/additional harbour basin (but <= 1 in total) (+ 0 or >=1 channel/Dredge/Marine structure)
(R3) – High to very high pressure	• ≥1 harbour basin + 0 Channel/Dredging/Marine structure<br>• ≥1 harbour basin + ≥1 channel<br>• ≥1 harbour basin + ≥1 dredging<br>• ≥1 harbour basin + ≥1 marine structure	• ≥1 harbour basin + 0 Channel/Dredging/Marine structure<br>• ≥1 harbour basin + ≥1 channel<br>• ≥1 harbour basin + ≥1 dredging<br>• ≥1 harbour basin + ≥1 marine structure

**Total (all atolls)**						
		**2014-2016 (Number of islands)**				
		**R1**	**R2**	**R3**	**n.d.**	**Total**
**2004-2006**	**R1**	330	36	3		369
	**R2**	3	157	25		185
	**R3**	0	4	36		40
	**n.d.**				14	14
	**Total**	333	197	64	14	608
		**2014-2016**				
		**% of the atoll sample**				
		**R1**	**R2**	**R3**	**n.d.**	**Total**
**2004-2006**	**R1**	54.3	5.9	0.5		60.7
	**R2**	0.5	25.8	4.1		30.4
	**R3**	0.0	0.7	5.9		6.6
	**n.d.**				2.3	2.3
	**Total**	54.8	32.4	10.5	2.3	100.0

**Nb**	**%**	**Change in human pressure**
7	1.2	Decrease
523	86.0	Stability (no change)
64	10.5	Increase
14	2.3	n.d.
608	100.0	Total

Table C. Island type (T), based on the combination of shoreline type (S) with human pressure exerted on the island's reef (R)

**Island type (T) and level of disturbance of the island's natural adjustment capacity**		**Categories (S) + (R)**
T1	No disturbance	• (S1) + (R1)
T2	Low disturbance	• (S1) + (R2)<br>• (S2) + (R1)
T3	Moderate disturbance	• (S1) + (R3)<br>• (S2) + (R2)<br>• (S2) + (R3)
T4	High disturbance	• (S3) + (R1)<br>• (S3) + (R2)<br>• (S3) + (R3)
T5	Very high disturbance	• (S4) + (R1)<br>• (S4) + (R2)<br>• (S4) + (R3)<br>• (S5) + (R1)<br>• (S5) + (R2)<br>• (S5) + (R3)

	**Type 1**	**Type 2**	**Type 3**	**Type 4**	**Type 5**	**n.d.**
**2004-2006**	364	101	90	12	27	14
**2014-2016**	329	70	135	14	46	14

Table D. Change in island type (T) between 2004-2006 and 2014-2016 for all of the sample islands

		**2014-2016**						
		**T1**	**T2**	**T3**	**T4**	**T5**	**n.d.**	**Total (2004-2006)**
**2004-2006**	**T1**	327	21	15	1	0	-	364
	**T2**	2	49	43	2	5	-	101
	**T3**	0	0	75	3	12	-	90
	**T4**	0	0	0	7	5	-	12
	**T5**	0	0	2	1	24	-	27
	**n.d.**	-	-	-	-	-	14	14
	**Total (2014-2016)**	329	70	135	14	46	14	**608**

**Nb**	**%**	**Change in human pressure**
5	0.8	Decrease
482	79.3	Stability (no change)
107	17.6	Increase
14	2.3	n.d.
608	100.0	Total

Table E. Change in island type (T) between 2004-2006 and 2014-2016 for the inhabited islands only

		2014-2016						
		**T1**	**T2**	**T3**	**T4**	**T5**	**n.d.**	**Total (2004-2006)**
**2004-2006**	**T1**	0	0	6	0	0	-	6
	**T2**	0	1	24	1	0	-	26
	**T3**	0	0	43	3	11	-	57
	**T4**	0	0	0	3	5	-	8
	**T5**	0	0	0	1	6	-	7
	**n.d.**	-	-	-	-	-	3	3
	**Total (2014-2016)**	0	1	73	8	22	3	**107**

**Nb**	**%**	**Change in human pressure**
1	0.9	Decrease
53	49.5	Stability (no change)
50	46.7	Increase
3	2.8	n.d.
107	100.0	Total
